# Guanxinjing ameliorates coronary microvascular dysfunction in myocardial ischemia-reperfusion injury by alleviating inflammation and restoring endothelial function

**DOI:** 10.3389/fimmu.2026.1846953

**Published:** 2026-07-17

**Authors:** Yaoting Wang, Lan Li, Liang Jin, Shikai Zhan, Hai Ren, Jiao Zhao, Qiuru Wang, Yiqiong Wang, Shudong Lin, Jiaxing Xin, Yao Dai, Yifan Zhou, Ting Zhang, Qian Liu, Yunyun Lu, Yiqi Zhu, Jiangyue Wu, Jiayan Lin, Shujing Zhang, Ling Zhang

**Affiliations:** 1 College of Life Science, Zhejiang Chinese Medical University, Hangzhou, China; 2 School of Medical Technology and Information Engineering, Zhejiang Chinese Medical University, Hangzhou, Zhejiang, China; 3 Shanghai Key Laboratory of Compound Chinese Medicines, The Ministry of Education Key Laboratory for Standardization of Chinese Medicines, Institute of Chinese Materia Medica, Shanghai University of Traditional Chinese Medicine, Shanghai, China; 4 Research and Development Department, Baoding Traditional Chinese Medicine Pharmaceutical Co., Ltd., Baoding, China; 5 School of Pharmaceutical Science, Zhejiang Chinese Medical University, Hangzhou, China; 6 Innovation Institute for Artificial Intelligence in Medicine, Zhejiang University, Hangzhou, China

**Keywords:** endothelial function, GuanXinJing, inflammation, microcirculation, myocardial ischemia-reperfusion injury

## Abstract

**Background:**

Coronary microvascular dysfunction (CMD) secondary to myocardial ischemia-reperfusion injury (MIRI) is characterized by profound inflammatory activation and endothelial impairment. Guanxinjing compound (GXJC) is a clinical drug widely used for coronary heart disease and angina pectoris closely associated with coronary microvascular function. Its efficacy and mechanisms against MIRI-induced CMD remain elusive.

**Methods:**

The chemical profiling of GXJC was performed via UHPLC-Q-Exactive HRMS. Network pharmacology analysis (NPA) screened the core targets and key pathways of GXJC. *In vivo*, C57BL/6 mice received 7-day GXJC pretreatment before MIRI modeling, followed by comprehensive evaluation using Evans blue/TTC staining, Hematoxylin and eosin staining (H&E), apoptosis assessment by TUNEL staining, immunofluorescence, Western blotting, and ELISA. For *in vitro* studies, a TNF-α-induced inflammatory injury model in HUVECs was established to dissect the underlying mechanisms.

**Results:**

UHPLC-Q-Exactive HRMS analysis identified 256 bioactive compounds in GXJC. NPA predicted the TNF signaling pathway as a potential key mechanism through which GXJC may ameliorate MIRI. *In vivo*, GXJC treatment reduced myocardial infarct size and cardiomyocyte apoptosis while downregulating pro-inflammatory factors. Mechanistically, GXJC suppressed vascular inflammation by inhibiting IL-6, IL-1β, and CCL2, attenuated endothelial contractile and diastolic dysfunction by modulating the eNOS/NO/ET-1 axis, and alleviated endothelial injury and thrombosis by lowering FGL2 and p-selectin levels.

**Conclusion:**

Our study demonstrates that GXJC acts as a potential therapeutic agent for MIRI-induced myocardial injury and associated microvascular endothelial dysfunction through the dual modulation of inflammatory cascades and microvascular endothelial protection. These mechanistic insights support its future clinical development for microvascular complications following revascularization.

## Graphical Abstract

**Figure:**
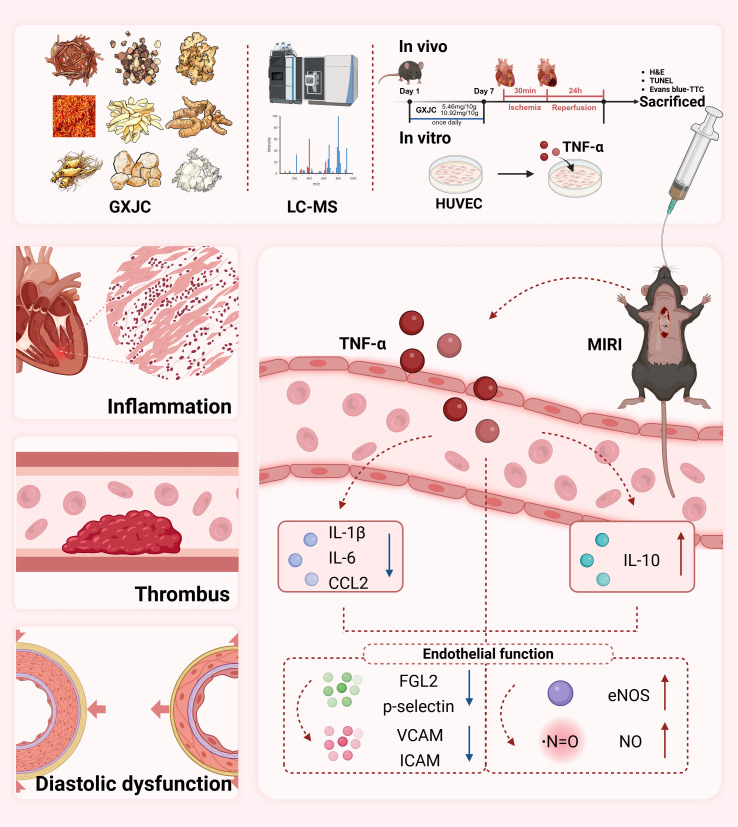

## 1 Introduction

Coronary heart disease (CHD) is a prevalent cardiovascular disorder, for which thrombolysis and percutaneous coronary intervention (PCI) remain the primary therapeutic approaches (1). However, these interventions may induce myocardial ischemia-reperfusion injury (MIRI), which subsequently contributes to coronary microvascular dysfunction (CMD) (2). CMD is characterized by inadequate myocardial perfusion or reduced coronary flow reserve resulting from structural and functional abnormalities of coronary microcirculation, ultimately leading to myocardial ischemia (3). The pathogenesis of CMD involves complex interactions between inflammatory cascades and endothelial cell dysfunction (4, 5). Endothelial dysfunction is driven by multiple interrelated pathological mechanisms, such as chronic low-grade inflammation, excessive oxidative stress, impaired vasomotor regulation, dysregulated neurohumoral control, reperfusion-induced endothelial injury, platelet hyperactivation, and a prothrombotic tendency (6–12).

Fernández-Sánchez et al. demonstrated that obesity induces upregulation of interleukin-6 (IL-6), tumor necrosis factor-α (TNF-α), and reactive oxygen species (ROS), but downregulation of nitric oxide (NO) bioavailability, and elevates the endothelium-derived contracting factor endothelin-1 (ET-1), eventually leading to endothelial dysfunction (13). Similarly, Tsai et al. revealed that inflammatory oxidative stress, accompanied by enhanced ET-1-mediated vasoconstriction, impairs the endothelial NO signaling pathway, ultimately contributing to myocardial hypertrophy, CMD, and ischemia (14).

Current therapies for CHD and angina pectoris mainly include three pathways: antiplatelet drugs, (e.g., ticagrelor), calcium channel blockers (e.g., diltiazem), and metabolic modulators (e.g., trimetazidine). However, they all present significant limitations. For instance, ticagrelor, a potent P2Y12 receptor antagonist, exerts antiplatelet effects by inhibiting adenosine diphosphate (ADP)-mediated platelet activation (15). However, its clinical application is limited by a risk of bleeding and dose-dependent dyspnea (16). Diltiazem, a calcium channel blocker, induces vasodilation by inhibiting Ca²^+^ influx (17). Nevertheless, its use may be associated with adverse effects, including hypotension, bradycardia, headache, nausea, and somnolence (18). Trimetazidine dihydrochloride, a piperazine derivative, modulates myocardial energy metabolism by selective inhibition of 3-ketoacyl-CoA thiolase, thereby shifting substrate utilization from fatty acid β-oxidation to glucose oxidation (19). Importantly, trimetazidine may induce or exacerbate movement disorders such as reversible parkinsonism, tremor, and orofacial dyskinesia (20). Therefore, it is necessary for an urgent clinical need to develop novel therapeutic strategies for CMD with well-defined efficacy and improved safety profiles.

Traditional Chinese Medicine (TCM) has emerged as a promising therapeutic approach for CMD through its unique multi-target and multi-pathway mechanisms, particularly via its synergistic anti-inflammatory, antioxidant, and hemodynamic regulatory effects. Accumulating evidence supports the therapeutic potential of TCM formulations in CMD. For example, Compound Danshen Dripping Pills can attenuate microvascular impairment through downregulating FOXO1 and reducing leukocyte adhesion (21). Gelanxinning Capsule restores endothelial function by its anti-inflammatory effects (22), and Tongmai Yangxin Pill activates PI3K/Akt/eNOS signaling to enhance NO-mediated vasodilation (23).

Guanxinjing compound (GXJC) is a clinically approved Chinese herbal compound preparation for the treatment of coronary heart disease, with National Medical Products Administration approval numbers Z20025812 and Z13021920. The therapeutic efficacy of GXJC depends not only on the inherent active components of its nine constituent herbs but also on the transformation of chemical constituents during compound processing. Compared with unprocessed raw herbs, GXJC exhibits significant changes in chemical composition after standardized processing. These transformed and interacting components may reduce toxicity and enhance efficacy, thereby providing a unique pharmacodynamic material basis for the multi-target regulation of MIRI-induced CMD. For instance, 5-hydroxymethylfurfural, a common reaction product generated during the heat processing of Chinese herbs, has been reported to alleviate inflammation by inhibiting the NF-κB signaling pathway and NLRP3 inflammasome activation (24). In addition, Salvia miltiorrhiza derivatives exert anti-inflammatory/antioxidant activities (25), though the specific mechanisms by which GXJC protects against MIRI-induced CMD remain elusive.

To systematically investigate the pharmacodynamic components of GXJC after processing and elucidate its mechanisms in treating MIRI-induced CMD, we conducted an integrated study combining chemical profiling, network pharmacology analysis (NPA), and experimental validation. First, the chemical constituents of GXJC were identified using UHPLC-Q-Exactive HRMS, and the identified compounds were subjected to NPA to predict potential targets and pathways. Second, a well-established murine MIRI model was used for *in vivo* validation to evaluate the cardioprotective efficacy of GXJC and its effects on microvascular function. Third, TNF-α-stimulated human umbilical vein endothelial cells (HUVECs) were employed for *in vitro* mechanistic studies to elucidate the endothelial protective pathways at the cellular and molecular levels. Our findings demonstrate that GXJC attenuates MIRI-induced myocardial injury and ameliorates associated microvascular endothelial dysfunction by suppressing inflammatory responses and restoring endothelial homeostasis.

## 2 Materials and methods

### 2.1 Materials and reagents

GXJC was obtained from Baoding Traditional Chinese Medicine Pharmaceutical Co., Ltd. (Baoding, Hebei, China) with the product batch number GXJJN2305005. Trimetazidine dihydrochloride (CAS: 13171-25-0) was obtained from Shanghai Bopharm Chemical Technology Co., Ltd. (Shanghai, China). TNF-α (HY-7058) was obtained from MedChemExpress (Monmouth Junction, NJ, USA). Evans blue (R31046) was obtained from Shanghai Yuanye Biotechnology Co., Ltd. (Shanghai, China). 2,3,5-Triphenyltetrazolium chloride (TTC; G3005) was obtained from Beijing Solarbio Science & Technology Co., Ltd. (Beijing, China). iF647-Tyramide (G1232), iF594-Tyramide (G1242), and iF488-Tyramide (G1231) were obtained from Wuhan Servicebio Technology Co., Ltd. (Wuhan, Hubei, China). Methanol and Acetonitrile (LC-MS Grade) were obtained from Tedia Co., Ltd (USA). Formic Acid (Chromatographic Grade) was obtained from Shanghai Aladdin Reagent Co., Ltd (Shanghai, China).

### 2.2 UHPLC-Q-exactive HRMS analysis of GXJC

The HPLC-MS analysis was performed using a Thermo U3000 UHPLC system coupled with a Q-Exactive high-resolution mass spectrometer. Chromatographic separation was achieved on a Welch Ultimate XB-C18 column (150 mm × 4.0 mm, 3 μm) with a mobile phase consisting of 0.1% formic acid in water (A) and acetonitrile (B). The gradient elution program was set as follows: 0–3 min at 2% B (isocratic), 3–5 min from 2% to 8% B, 5–24 min from 8% to 22% B, 24–30 min from 22% to 45% B, 30–35 min from 45% to 50% B, 35–40 min from 50% to 60% B, 40–42 min from 60% to 90% B, and 42–45 min at 90% B (isocratic). The flow rate was maintained at 0.4 mL/min, the column temperature was set at 35°C, and the injection volume was 5 μL. Mass spectrometric detection was conducted using a Q-Exactive instrument equipped with a HESI source operating in both positive and negative ionization modes. Key MS parameters were configured as follows: scan mode: Full MS/dd-MS², spray voltage: 2.5 kV, sheath gas flow rate: 50 arb, auxiliary gas flow rate: 14 arb, ion transfer tube temperature: 300°C, probe heater temperature: 500°C, mass scan range: *m/z* 100-1500, and HCD collision energies: 20, 30, and 40 eV. Data acquisition was controlled by Xcalibur 2.3.1 software. Raw data were processed using Compound Discoverer 3.1 software with a self-built compound library and workflow incorporating compound information from public databases including PubChem, CAS SciFinder, and CNKI. Processing parameters included a retention time range of 0-47 min, mass range of 100–1500 Da, positive ion adducts ([M+H]^+^, [M+Na]^+^, [M+K]^+^), negative ion adducts ([M-H]^-^, [M+FA-H]^-^), mass error tolerance of 5.0 ppm, S/N threshold of 3, and minimum peak intensity of 10^6^ for both ionization modes, enabling comprehensive peak extraction, normalization, and compound annotation.

### 2.3 Network pharmacological analysis

The chemical composition of GXJC was analyzed using a UHPLC-Q-Exactive HRMS mass spectrometer, and the obtained compound structure data were derived from the Chemspider database. Potential targets were forecasted using SwissTargetPrediction, retaining entries with probability > 0. Genes associated with MIRI were identified by searching the Genecards database (26) (https://www.genecards.org/), the OMIM database (27) (https://www.omim.org/), and the TTD database (28) (https://db.idrblab.net/ttd). Duplicate genes retrieved from these three databases were merged to eliminate redundancy, generating a consolidated set of MIRI-related target genes. The Bioinformatics online platform (https://www.bioinformatics.com.cn/) was employed to generate a Venn diagram visualizing the intersection between GXJC targets and MIRI targets.

The drug-disease intersection targets were imported into the STRING database (https://cn.string-db.org/) to construct a protein-protein interaction (PPI) network. This PPI network was then imported into Cytoscape 3.10.0 software. Network topology analysis was performed using the CytoNCA plugin, applying the following centrality measures to identify key nodes: Degree Centrality (DC), Closeness Centrality (CC), Betweenness Centrality (BC), Network Centrality (NC), Eigenvector Centrality (EC), and Local Average Connectivity-based method (LAC). Targets scoring above the median value for each centrality measure were considered key targets. The core targets identified from the PPI network analysis were imported back into Cytoscape 3.10.0. A network visualization was generated to illustrate the interactions between GXJC, its active components, and their corresponding targets.

Functional enrichment analysis of the potential targets was conducted using the DAVID bioinformatics annotation platform (https://davidbioinformatics.nih.gov/). This included Kyoto Encyclopedia of Genes and Genomes (KEGG) pathway enrichment analysis and Gene Ontology (GO) enrichment analysis.

### 2.4 Animals preparation

Fifty 6-week-old male C57BL/6 mice (18–20 g) were purchased from Shanghai SLAC Laboratory Animal Co., Ltd. All animal procedures and experimental protocols were approved by the Animal Ethics and Welfare Committee of Zhejiang Chinese Medical University (Approval No.: IACUC-20240930-26; Ethics Application ID: 202409-0927) and conducted in accordance with the National Institutes of Health (NIH) Guide for the Care and Use of Laboratory Animals. Following a one-week acclimatization period under specific pathogen-free (SPF) facility conditions (23 ± 2°C, 50% relative humidity, 12 h light/dark cycle) with free access to food and water, the mice were randomly divided into five groups (n=10 per group): Sham group, MIRI model group, Trimetazidine group (TMZ, 32 mg/kg/d), GXJC low-dose group (GXJC-L, 0.546 g/kg/d), and GXJC high-dose group (GXJC-H, 1.092 g/kg/d). The GXJC doses were determined based on the clinical human dose (0.06 g/kg) and the body surface area conversion factor (9.1 for human to mouse) (29). Accordingly, the low dose (0.546 g/kg/d) was calculated as the mouse equivalent of the clinical dose, and the high dose (1.092 g/kg/d) was set as twice the low dose.

### 2.5 Establishment of MIRI model

Drugs were administered via oral gavage once daily for 7 consecutive days prior to surgery, with a gavage volume of 0.1 mL/10 g body weight. The treatment groups (GXJC-L, GXJC-H, and TMZ) received their respective compounds, while the Sham and MIRI model groups received an equal volume of 0.9% saline solution as the vehicle control. Following the final gavage administration, mice underwent MIRI surgery. Briefly, mice were anesthetized with sodium pentobarbital (50 mg/kg, i.p.). After tracheal intubation, animals were mechanically ventilated. Mice were placed on a warming pad to maintain body temperature at 37.0 ± 0.5 °C. The heart was exposed via a left thoracotomy. The left anterior descending coronary artery (LAD) was ligated for 30 minutes using a 7–0 silk suture. Successful induction of myocardial ischemia was confirmed by persistent ST-segment elevation on electrocardiogram monitoring and visual observation of pallor in the myocardium distal to the ligation site. After 30 minutes of ischemia, the ligature was released to initiate reperfusion. The chest cavity was closed in layers. Following 24 h of reperfusion, the mice were deeply anesthetized with pentobarbital sodium (50 mg/kg, i.p.), and euthanized by cervical dislocation prior to tissue collection. Serum and cardiac tissue samples were collected for subsequent analysis. A total of 10 animals were subjected to surgery in each group. To accommodate the distinct tissue preparation requirements for various assays, the animals were allocated into two subgroups. Five animals were designated for infarct size assessment using Evans blue - TTC double staining. The remaining five animals were used for serum collection and histological/molecular analyses.

### 2.6 Evans blue - TTC double staining

Myocardial infarct size was assessed using Evans blue and TTC double staining. After 24 h of reperfusion, hearts were immediately excised, rinsed, and subjected to retrograde perfusion via the aortic root with 0.2 mL of 1% Evans blue dye. Following a wash with phosphate-buffered saline (PBS), hearts were frozen at -20 °C for 30 minutes. Hearts were then transversely sectioned into 1-mm thick slices from apex to base using a heart matrix, yielding 5–6 slices per heart. The slices were immersed in TTC staining solution and incubated at 37 °C for 20 minutes. Subsequently, slices were fixed in 4% paraformaldehyde solution (PFA) at room temperature for 24 h. Stained myocardial sections were photographed. Non-ischemic areas appeared blue (Evans blue-perfused), the area at risk (AAR) stained red (viable myocardium), and the infarcted area (INF) remained white (necrotic, TTC-negative).The left ventricular area (LV), AAR, and INF were quantified using ImageJ software. The AAR/LV ratio (%) represents the ischemic risk zone, and the INF/AAR ratio (%) reflects the actual infarct size within the risk zone.

### 2.7 Hematoxylin and eosin staining

Cardiac tissue samples were fixed in 4% PFA at room temperature for 24 hours. Subsequently, tissues were dehydrated through a graded ethanol series, cleared in xylene, and embedded in paraffin blocks. Sections of 4-μm thick were cut from the paraffin blocks using a rotary microtome, mounted onto glass slides, and allowed to dry overnight. Following deparaffinization, sections were stained with hematoxylin and eosin (H&E). Histopathological evaluation was performed using a light microscope, and digital images of all specimens were systematically recorded.

### 2.8 Immunofluorescence staining

As described previously, heart tissue sections underwent antigen retrieval by immersion in preheated (95 °C) antigen retrieval solution (1×) for 20 minutes. After cooling to room temperature, sections were blocked with 5% bovine serum albumin (BSA) for 30 minutes. Primary antibodies were applied and incubated overnight at 4 °C. The following primary antibodies were used: α-SMA (ET1607-53, huabio, Hangzhou, China), CD31 (ER31219, huabio), FGL2 (11827-1-AP, Proteintech, Wuhan, China), and ET-1 (12191-1-AP, Proteintech). The following day, sections were washed three times with PBS (5 minutes per wash). Subsequently, sections were incubated with corresponding horseradish peroxidase (HRP)-conjugated secondary antibodies for 1 hour at room temperature under light-protected conditions. After three additional PBS washes (5 minutes each), signal amplification was performed using Tyramide Signal Amplification (TSA). The above protocol (primary antibody incubation, washing, secondary antibody incubation, washing, TSA) was then repeated for Cardiac Troponin I (cTnI; ET1702-37, huabio) under light-protected conditions. Nuclei were counterstained with DAPI (G1012, Servicebio, Wuhan, China). Sections were mounted using anti-fade mounting medium (P0126, Beyotime, Shanghai, China). Images of each section were captured using a fluorescence inverted microscope (ZEISS, Germany). Quantitative analysis of fluorescence signals was performed.

### 2.9 Terminal deoxynucleotidyl transferase dUTP nick end labeling staining

Cardiomyocyte apoptosis was assessed using the TMR (red) TUNEL Cell Apoptosis Detection Kit (G1502, Servicebio, Wuhan, China) according to the manufacturer’s instructions. Briefly, tissue sections were permeabilized and incubated with the TUNEL reaction mixture to label DNA fragmentation. To specifically identify apoptotic cardiomyocytes, sections were subsequently immunostained with an antibody against cTnI under light-protected conditions, followed by DAPI counterstaining for nuclear visualization. Fluorescence images were captured using a fluorescence inverted microscope. The apoptotic level was quantified by measuring the fluorescence intensity of TUNEL staining specifically within cTnI^+^ areas, using ImageJ software (NIH, USA).

### 2.10 Nitric oxide measurement

The total NO production in serum was quantified by measuring the concentrations of its stable metabolites (nitrate and nitrite) using a modified Griess reaction assay, following the manufacturer’s protocol for the NO Assay Kit (S0021S, Beyotime). Optical density was determined at 540 nm, and sodium nitrite was employed as a reference standard to calculate nitrite levels in serum samples. Statistical analyses were performed on the resulting data.

### 2.11 Enzyme-linked immunosorbent assay

Cellular protein levels of ICAM-1 (EH0064, Youke Biotech, Hangzhou, China), IL-6 (EH0001, Youke Biotech), and CCL2/MCP-1 (EHC113, NeoBioscience, Shenzhen, China) in HUVECs supernatants were quantified using commercial ELISA kits according to manufacturers’ protocols. HUVECs were seeded in 6-well plates and treated with TNF-α and GXJC (0.75, 1.0, and 1.5 mg/mL) at 60-70% confluency for 24 h. Supernatants were collected, centrifuged at 2,000 × g for 5 min, and stored at -80 °C prior to analysis.

Serum levels of IL-1β (SPS-23186, Saipaishen Biology, Shanghai, China), IL-10 (SPS-23174, Saipaishen Biology), IL-6 (SPS-23200, Saipaishen Biology), and CCL2/MCP-1 (EMC113, NeoBioscience) from mice were similarly assessed. Absorbance measurements were converted to concentrations using standard curves generated by Four-Parameter Logistic (4PL) regression. Statistical analysis was performed on concentration data.

### 2.12 Cell culture

Human Umbilical Vein Endothelial Cells (HUVECs) were purchased from Zhejiang BaiDi Biotechnology Co., Ltd. (Hangzhou, China). Cells were cultured in low-glucose Dulbecco’s Modified Eagle’s Medium (DMEM, C11885500BT, Gibco, Germany), supplemented with 10% fetal bovine serum (FBS, C04001, VivaCell, Shanghai, China) and 1% antibiotic-antimycotic (C100C8, NCM Biotech, Suzhou, China). HUVECs were maintained at 37 °C in a humidified incubator (Thermo Fisher Scientific, USA) with 5% CO_2_. Based on preliminary CCK-8 cell viability and Phalloidin cytoskeletal integrity assays, GXJC concentrations of 0.75, 1.0, and 1.5 mg/mL were selected for subsequent experiments to ensure optimal pharmacological efficacy without inducing cellular toxicity.

### 2.13 Cell counting kit-8 assay

Cell viability and GXJC cytotoxicity were assessed using a CCK-8 (C0038, Beyotime, Shanghai, China) according to the manufacturer’s protocol. HUVECs were treated with varying concentrations of GXJC for 24 h. Briefly, cells were seeded in 96-well plates at 1×10^5^ cells/mL. After drug treatment, 20 µL CCK-8 reagent was added to each well containing 200 µL culture medium and incubated for 2 hours at 37°C. Absorbance at 450 nm was measured using an EnSpire multimode plate reader (PerkinElmer, USA). Viability data were normalized to vehicle-treated controls.

### 2.14 Phalloidin staining

Cells were cultured in confocal dishes as previously described. Following three washes with PBS, cells were fixed with 4% PFA at 4 °C for 20 minutes and subsequently washed with PBS. Permeabilization was performed by incubation in 0.5% Triton X-100 for 10 minutes, followed by additional PBS washes. F-actin was stained with phalloidin (CA1620, Solarbio, Beijing, China) for 40 minutes at room temperature under light-protected conditions. After three PBS washes, nuclei were counterstained with DAPI for 10 minutes at room temperature, followed by three final PBS washes. Fluorescence images were acquired using a laser scanning confocal microscope (ZEISS, Germany) and digitally archived.

### 2.15 Reverse transcription-quantitative polymerase chain reaction

Total RNA was extracted from HUVECs using an RNA isolation kit (Proteintech, Wuhan, China) according to the manufacturer’s protocol. cDNA synthesis was performed with a First Strand cDNA Synthesis Kit (Seq-Hunt, Beijing, China). cDNA amplification was carried out using a real-time PCR system (Bio-Rad, USA) with a three-step cycling protocol: initial denaturation at 95 °C for 5 min; followed by 40 cycles of denaturation at 95 °C for 10 s, annealing at 60 °C for 20 s, and extension at 72 °C for 20 s. Gene-specific primers for mRNA detection (synthesized by Shangya Biotechnology, Zhejiang, China) are listed in **Supplementary Table 1**. Target gene expression was normalized to β-actin and calculated using the 2^−ΔΔCt^ method.

### 2.16 Western blotting

Total protein was extracted from cardiac tissues or HUVECs using Cell Lysis Buffer for Western and IP (P0013, Beyotime, Shanghai, China) supplemented with Protease and Phosphatase Inhibitor Cocktail (P002, NCM Biotech, Suzhou, China). Protein samples were separated by sodium dodecyl sulfate-polyacrylamide gel electrophoresis (SDS-PAGE) and electrophoretically transferred to polyvinylidene difluoride (PVDF) membranes. Membranes were blocked with 5% non-fat milk for 1 h at room temperature. Subsequently, membranes were incubated overnight at 4 °C with gentle agitation in primary antibodies diluted in blocking buffer. Primary antibodies included: IL-6 (A0286, Abclonal, Wuhan, China), IL-1β (26048-1-AP, Proteintech, Wuhan, China), FGL2 (11827-1-AP, Proteintech), ET-1 (12191-1-AP, Proteintech), VEGF (ER30607, huabio), eNOS (AF0096, Affinity Biosciences, Jiangsu, China). The following day, following three 10-minute TBST washes, membranes were incubated with appropriate horseradish peroxidase (HRP)-conjugated species-specific secondary antibodies for 1 h at room temperature. After additional TBST washes, protein bands were visualized using enhanced chemiluminescence (ECL) substrate (P10200, NCM Biotech) and imaged with a ProteinSimple FluorChem imaging system. Relative protein expression levels were quantified using ImageJ software and normalized to β-actin loading controls.

### 2.17 Molecular docking

Molecular docking was performed using AutoDock 4.2.6 to investigate the binding affinity between the active ingredients and key targets. The top 10 compounds from GXJC, screened based on PPI network degree values, were retrieved from PubChem as ligands. The crystal structures of ET-1, eNOS, and TNF were obtained from the RCSB Protein Data Bank (PDB) as receptors. Both ligands and proteins were prepared using AutoDock Tools, including the addition of polar hydrogens and Gasteiger charges. The Lamarckian Genetic Algorithm (LGA) was employed for the docking simulations. The binding affinity was assessed based on the binding energy (kcal/mol), where a lower value indicates stronger binding potential. Finally, the optimal docking conformations were visualized.

### 2.18 Statistical analysis

All statistical analyses were performed using GraphPad Prism software (version 9.0, GraphPad Software, San Diego, CA, USA). Prior to hypothesis testing, all continuous datasets were evaluated for normal distribution using the Shapiro-Wilk test and for homogeneity of variance using the Brown-Forsythe test. For multiple group comparisons, a one-way analysis of variance (ANOVA) followed by Dunnett’s multiple comparisons test was conducted only when data satisfied both assumptions of normality and homogeneity of variance. If the data violated the assumption of normality or homogeneity of variance, the non-parametric Kruskal-Wallis test followed by Dunn’s multiple comparisons test was performed. Normally distributed data with equal variances are expressed as mean with standard error of the mean (SEM), whereas data violating these assumptions are presented as the median with interquartile range (IQR). Statistical significance was defined as a two-tailed *P* < 0.05.

## 3 Results

### 3.1 Chemical profiling of GXJC and NPA of its mechanisms against MIRI

**Figure 1:**
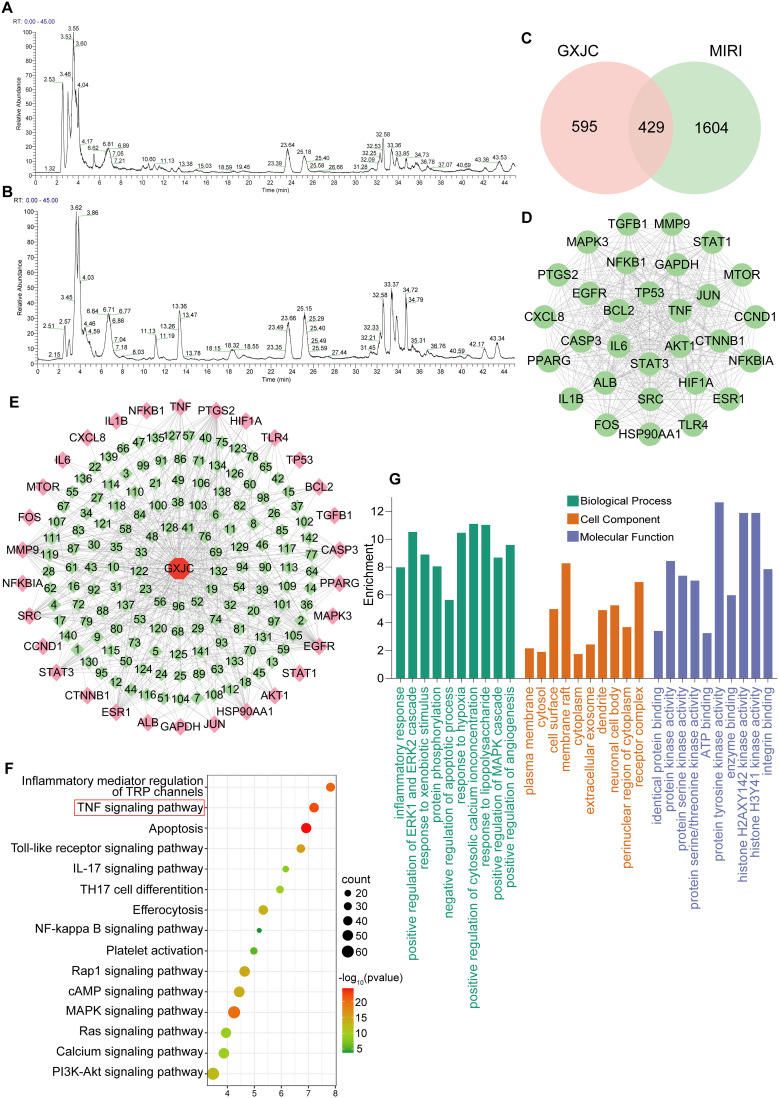
Network pharmacology diagram of GXJC for the treatment of MIRI. **(A)** The total Ion Chromatogram of GXJC at positive mode by UHPLC-Q-Exactive HRMS. **(B)** The total Ion Chromatogram of GXJC at negative mode by UHPLC-Q-Exactive HRMS. **(C)** Venn diagram of the drug targets of GXJC and the disease targets of MIRI. **(D)** The PPI networks after three rounds of screening based on topological algorithms (DC, CC, BC, NC, EC, LAC). **(E)** The herb-compound-Targets gene network constructed using core genes and corresponding compounds. Detailed compound information is available in **Supplementary Table 3**. **(F)** KEGG Pathways. **(G)** GO terms, including biological process (BP), cellular component (CC), and molecular function (MF).

To investigate the chemical components of GXJC, UHPLC-Q-Exactive HRMS was performed in both positive and negative ionization modes. The corresponding total ion chromatograms are shown in **Figures 1A, B** and the base peak chromatograms are shown in **Supplementary Figure S1A, B**. The chemical composition of GXJC extract was determined by comparing chromatographic retention times and fragment ion peaks, leading to the identification of 256 major chemical constituents. Detailed information on these compounds is provided in **Supplementary Table 2**.

MIRI-related targets were collected from GeneCards, OMIM, and TTD databases, yielding a total of 2,033 genes. Venn diagram analysis using the Bioinformatic Cloud Platform identified 429 overlapping targets between GXJC-related targets and MIRI-related targets (**Figure 1C**). These overlapping targets were then imported into the STRING database to construct a PPI network, comprising 428 nodes and 11232 edges (**Supplementary Figure S2A**). The PPI network was further analyzed in Cytoscape using the CytoNCA plugin. Key targets were identified through successive filtering based on centrality metrics, including degree centrality (DC), closeness centrality (CC), betweenness centrality (BC), network centrality (NC), eigenvector centrality (EC), and local average connectivity (LAC). The first two filtering rounds yielded 151 and 69 potential core targets, respectively (**Supplementary Figure S2B, C**), and the third round further refined these candidates to 30 high-confidence core targets (**Figure 1D**).

Next, these 30 core targets and their corresponding active compounds were used to construct a compound-target-pathway network in Cytoscape (**Figure 1E**; **Supplementary Table 3**). KEGG pathway enrichment analysis and Gene Ontology (GO) enrichment analysis were then performed using the DAVID database, with significantly enriched terms screened using the criterion FDR < 0.05. KEGG enrichment analysis identified 193 significantly enriched pathways, including inflammatory mediator regulation of TRP channels, TNF signaling pathway, and apoptosis (**Figure 1F**). GO enrichment analysis identified 757 biological process (BP) terms, including inflammatory response and negative regulation of apoptotic process; 108 cellular component (CC) terms, including membrane raft and receptor complex; and 208 molecular function (MF) terms, including protein kinase activity and protein tyrosine kinase activity (**Figure 1G**). Collectively, these findings indicate that GXJC ameliorates MIRI-induced CMD by modulating inflammatory cytokines, including IL-6, IL-1β, and TNF, and by targeting the TNF signaling pathway to attenuate myocardial inflammation.

### 3.2 GXJC protects against MIRI-induced myocardial injury by reducing infarct size and suppressing apoptosis

**Figure 2:**
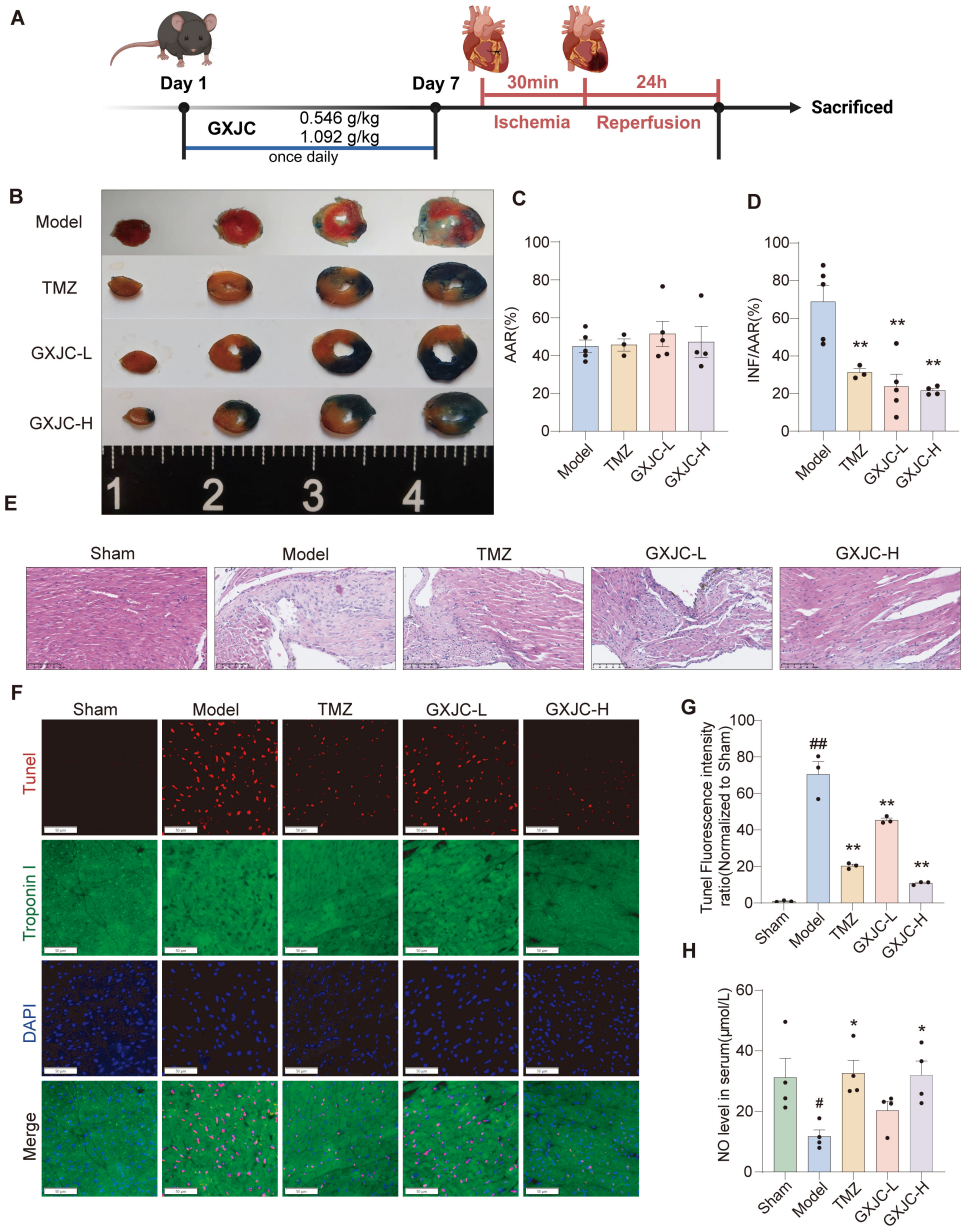
GXJC attenuates MIRI-induced myocardial injury in mice. **(A)** Flowchart of Animal Experiments and Establishment of the MIRI Model. **(B)** The area of myocardial infarction was detected by Evans blue-TTC Double Staining. The nonischemic section shown in blue area, red represent risk area, and the infarct region is stained white. **(C, D)** The INF/AAR and AAR/LV ratios were quantified (n ≥ 3). **(E)** H&E staining of myocardial tissue sections, scale bar = 100 μm. **(F, G)** Apoptosis was evaluated via TUNEL staining and quantitative analysis of cardiomyocyte apoptosis by measuring the TUNEL fluorescence intensity within cTnI^+^ areas (green: cTnI, blue: DAPI-stained nuclei, red: TUNEL, scale bar = 50 μm) (n = 3). **(H)** NO content in the serum, measured by the Griess assay (n = 4). ^#^*P* < 0.05, ^##^*P* < 0.01 vs. Sham group. ^*^P < 0.05, ^**^P < 0.01 vs. Model group.

To evaluate the protective effect of GXJC against MIRI-induced CMD, mice were intragastrically administered either GXJC (GXJC-L: 0.546 g/kg; GXJC-H: 1.092 g/kg) or trimetazidine (TMZ, 32 mg/kg) once daily for 7 consecutive days. After treatment, the MIRI model was established by ligating the LAD for 30 min of ischemia, followed by 24 h of reperfusion (**Figure 2A**). TTC/Evans blue staining revealed that both GXJC-H and TMZ significantly reduced the infarct size relative to the area at risk (INF/AAR),whereas no significant differences were observed in the area at risk relative to the left ventricle (AAR/LV) among the groups (**Figures 2B–D**). H&E staining demonstrated well-organized myocardial fibers with centrally located nuclei and no inflammatory infiltration in the Sham group. In contrast, the Model group exhibited marked inflammatory infiltration and disrupted cardiomyocyte arrangement, while GXJC-H significantly attenuated these pathological changes (**Figure 2E**). TUNEL staining was performed to assess cardiomyocyte apoptosis 24 h after reperfusion. The apoptotic level in cardiomyocytes, evaluated by TUNEL fluorescence intensity within cTnI^+^ areas, was significantly elevated in the Model group compared with the Sham group, whereas GXJC-H and TMZ markedly suppressed MIRI-induced apoptosis. (**Figures 2F, G**). In addition, serum NO levels were significantly lower in the Model group compared with the Sham group (P = 0.0217). Treatment with both GXJC-H and TMZ significantly restored serum NO levels compared to the Model group (both P < 0.05) (**Figure 2H**). Collectively, these findings demonstrate that GXJC, particularly at the high dose, protects against MIRI-induced myocardial injury by reducing infarct size, suppressing apoptosis, alleviating inflammatory infiltration, and improving NO bioavailability in mice with MIRI-induced CMD.

### 3.3 GXJC alleviates MIRI-induced inflammation

**Figure 3:**
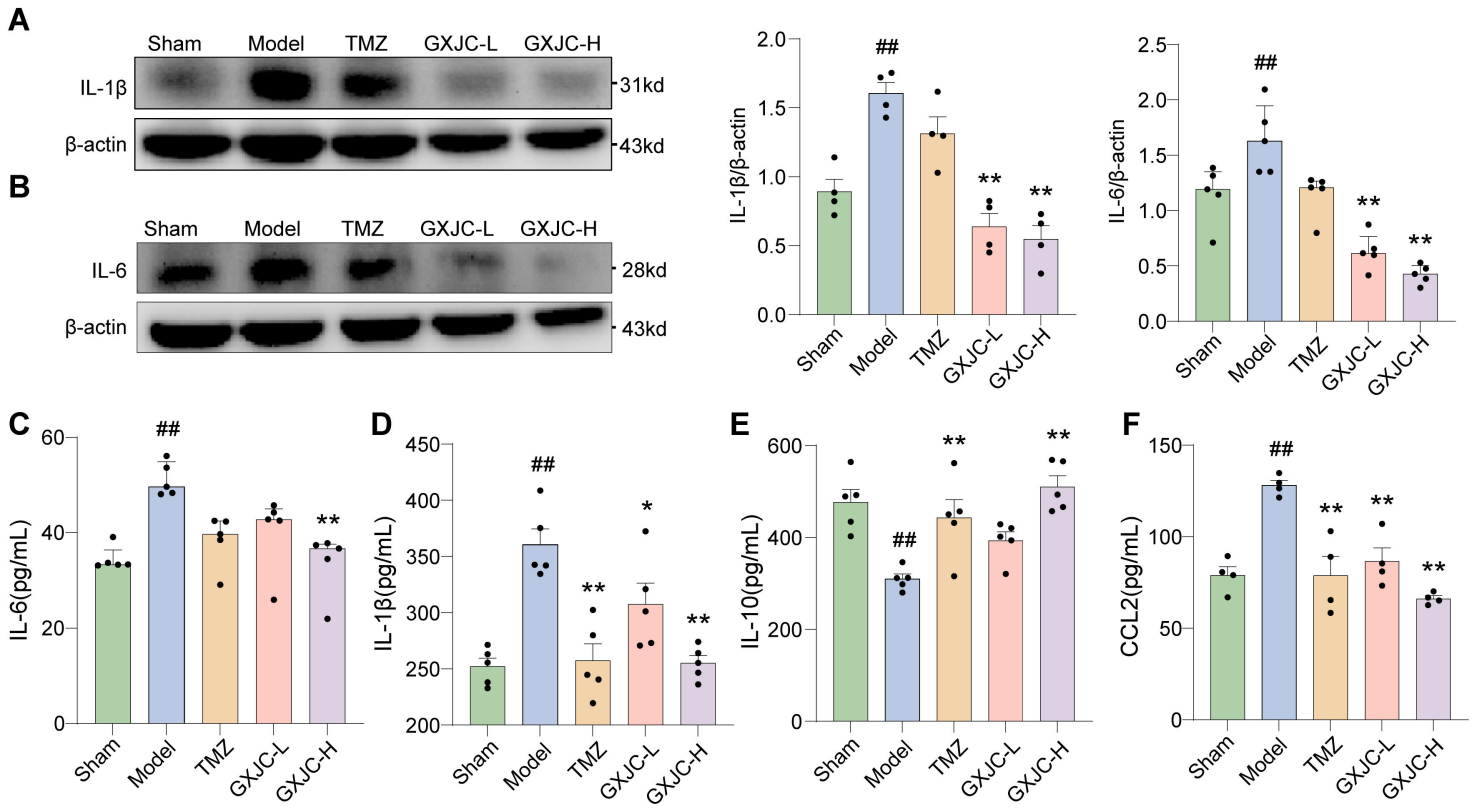
GXJC alleviates MIRI-induced inflammation. A-B The expression of IL-1β **(A)**, IL-6 **(B)** were detected by WB, (n ≥ 4). C-F Protein levels of IL-6 **(C)**, IL-1β **(D)**, IL-10 **(E)**, and CCL2 **(F)** in serum, measured by ELISA (n≥4). ^#^*P* < 0.05, ^##^*P* < 0.01 vs. Sham group. ^*^P < 0.05, ^**^P < 0.01 vs. Model group.

Previous studies have shown that MIRI triggers inflammatory activation (30). To validate whether GXJC modulates inflammation after MIRI, Western blotting was performed to assess inflammatory protein in myocardial tissue. Compared with the Sham group, the Model group showed markedly increased protein levels of IL-1β and IL-6. Notably, GXJC treatment significantly suppressed the MIRI-induced upregulation of both IL-1β and IL-6 (**Figures 3A, B**). Consistently, ELISA results showed that GXJC significantly decreased the levels of the pro-inflammatory mediators IL-6, IL-1β, and CCL2, while increasing the level of the anti-inflammatory cytokine IL-10 (**Figures 3C–F**). Collectively, these findings indicate that GXJC attenuates MIRI-induced coronary microvascular inflammation by suppressing pro-inflammatory mediators, including IL-6, IL-1β, and CCL2, and enhancing the anti-inflammatory response.

### 3.4 GXJC ameliorates MIRI-induced endothelial dysfunction

**Figure 4:**
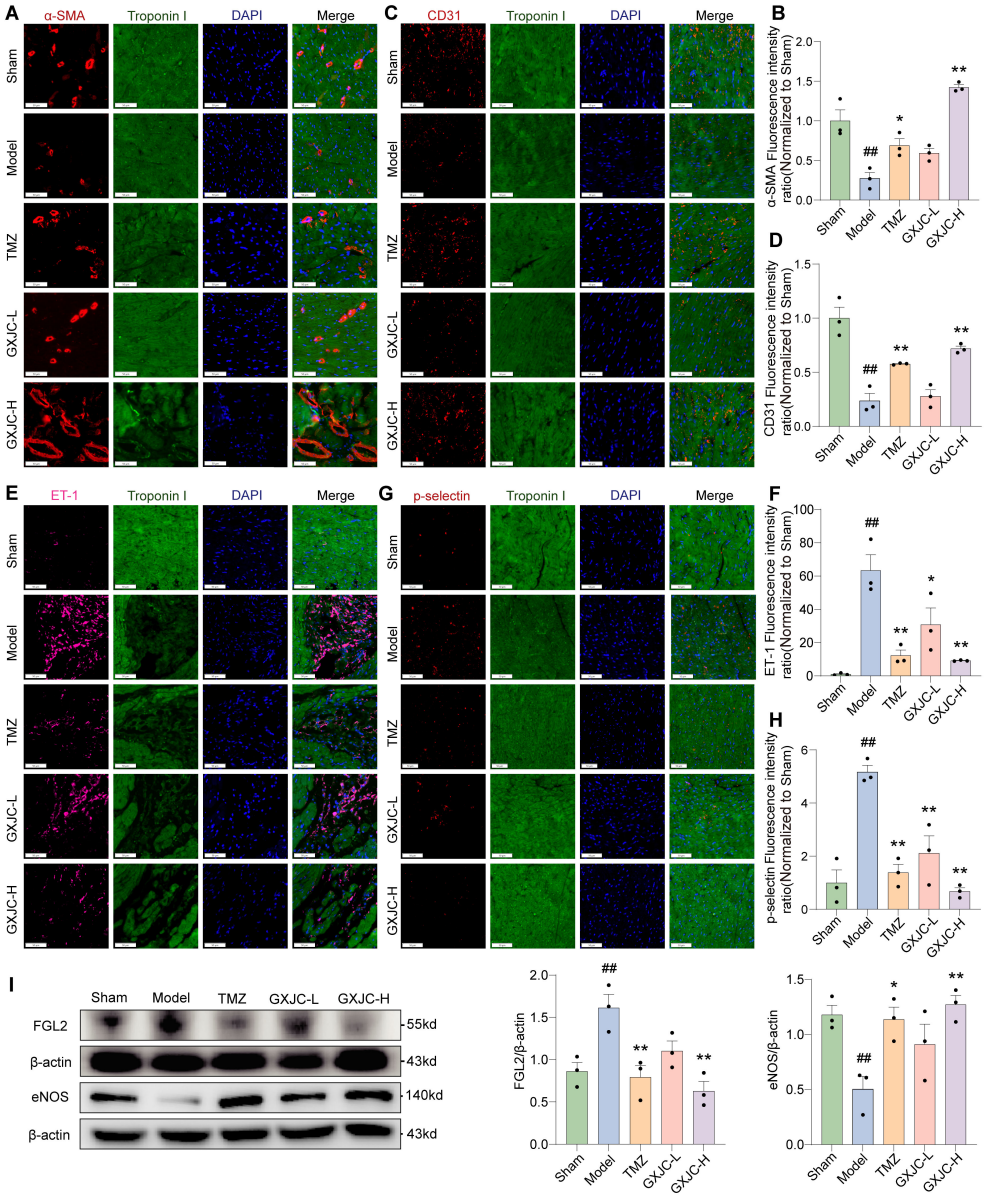
GXJC ameliorated vascular endothelial function following MIRI injury. **(A, B)** Representative immunofluorescence images of α-SMA in myocardial tissues and quantitative analysis, scale bar = 50 μm (n = 3). **(C, D)** Representative immunofluorescence images of CD31 in myocardial tissues and quantitative analysis, scale bar = 50 μm (n = 3). **(E, F)** Representative immunofluorescence images of ET-1 in myocardial tissues and quantitative analysis, scale bar = 50 μm (n = 3). **(G, H)** Representative IF images of p-selectin in myocardial tissues and quantitative analysis, scale bar = 50 μm (n = 3). **(I)** The expression of FGL2 and eNOS were detected by WB. (n = 3). ^#^*P* < 0.05, ^##^*P* < 0.01 vs. Sham group. ^*^P < 0.05, ^**^P < 0.01 vs. Model group.

Given the close association between inflammation and endothelial dysfunction, we evaluated the protective effects of GXJC on the vascular endothelium after MIRI. Immunofluorescence co-staining for α-SMA, CD31, and cTnI was performed to assess vascular integrity, endothelial injury. Compared with the Sham group, the Model group exhibited significantly reduced α-SMA and CD31 expression, indicating impaired vascular structure and endothelial damage. In contrast, treatment with TMZ or GXJC-H restored α-SMA and CD31 expression, suggesting that GXJC preserved vascular structural integrity and ameliorated MIRI-induced endothelial injury (**Figures 4A–D**). Furthermore, ET-1 and p-selectin expression were markedly elevated after MIRI, whereas GXJC treatment significantly reversed these changes (**Figures 4E–H**). Western blotting analysis further showed that FGL2 protein expression was significantly increased, while eNOS expression was decreased in the Model group compared with the Sham group. These alterations were similarly reversed by TMZ and GXJC-H treatment (**Figure 4I**). Collectively, these results demonstrate that GXJC protects against microvascular structural damage, improves endothelium-dependent vasoconstriction and vasodilation dysfunction, reduces inflammation-mediated thrombosis, and thereby alleviates endothelial dysfunction.

### 3.5 GXJC alleviates TNF-α-induced endothelial inflammation and counteracts cytoskeletal remodeling

**Figure 5:**
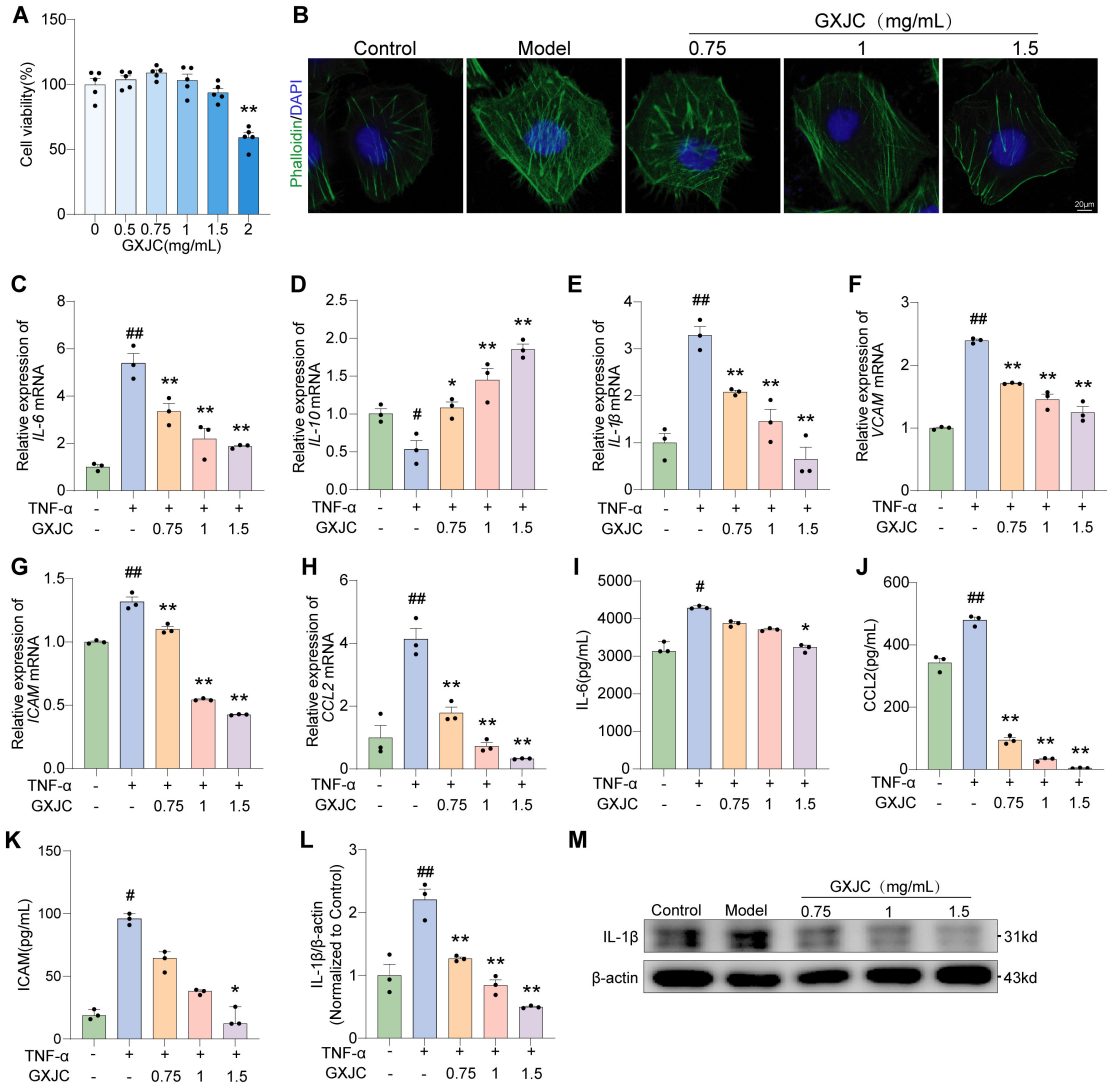
GXJC inhibited TNF-α-induced inflammation in HUVECs. **(A)** Cell viability of HUVECs treated with various concentrations of GXJC, assessed by CCK-8 assay (n=5). **(B)** Representative fluorescence images of the F-actin cytoskeleton in HUVECs stained with phalloidin. Images are representative of three independent experiments with similar results. Scale bar = 20 μm. **(C-H)** Relative mRNA expression levels of *IL-6*, *IL-10*, *IL-1β*, *ICAM-1*, *VCAM-1*, and *CCL2* in HUVECs, measured by RT-qPCR (n=3). I-K Protein levels of IL-6, CCL2, and ICAM-1 in HUVECs, measured by ELISA (n=3). L-M The expression of IL-1β were detected by WB in HUVECs (n = 3). ^#^*P* < 0.05, ^##^*P* < 0.01 vs. Control group. ^*^P < 0.05, ^**^P < 0.01 vs. Model group.

To further elucidate the effects of GXJC on endothelial cell function, we employed an *in vitro* inflammatory model by stimulating HUVECs with 10 ng/mL TNF-α, as previously described (31). We first assessed the impact of GXJC on HUVECs viability. As shown in **Figure 5A**, treatment with 2 mg/mL GXJC for 24 h exhibited cytotoxicity. Therefore, GXJC concentrations of 0.75, 1, and 1.5 mg/mL were selected for subsequent studies. Phalloidin staining was used to examine morphological changes in endothelial cells under inflammatory conditions and to determine the protective effect of GXJC on cytoskeletal organization. Control cells exhibited a regular polygonal or spindle-shaped morphology, with clear cell boundaries and fine, evenly distributed stress fibers throughout the cytoplasm. In contrast, TNF-α-induced inflamed endothelial cells exhibited irregular shapes, enlarged cell area, increased peripheral pseudopodia, and thickened, aggregated stress fibers, indicating marked cytoskeletal remodeling. These morphological alterations were substantially ameliorated by GXJC treatment (**Figure 5B**).

To further investigate the anti-inflammatory effects of GXJC, RT-qPCR was performed to assess inflammatory gene expression. GXJC significantly reduced TNF-α-induced mRNA expression of *IL-6*, *IL-1β*, *VCAM*, *ICAM*, and *CCL2*, while increasing *IL-10* mRNA expression (**Figures 5C–H**). Consistently, GXJC markedly inhibited the secretion of IL-6, CCL2, and ICAM proteins (**Figures 5I–K**). Western blotting analysis further showed that GXJC significantly decreased IL-1β protein expression (**Figures 5L, M**). Collectively, these results indicate that GXJC mitigates endothelial inflammation in HUVECs by suppressing levels of inflammatory cytokines and chemokines. Furthermore, GXJC counteracts cytoskeletal remodeling and improves cellular structure.

### 3.6 GXJC ameliorates endothelial dysfunction by restoring the eNOS/NO axis and suppressing the FGL2/ET-1 pathway in TNF-α-stimulated HUVECs

**Figure 6:**
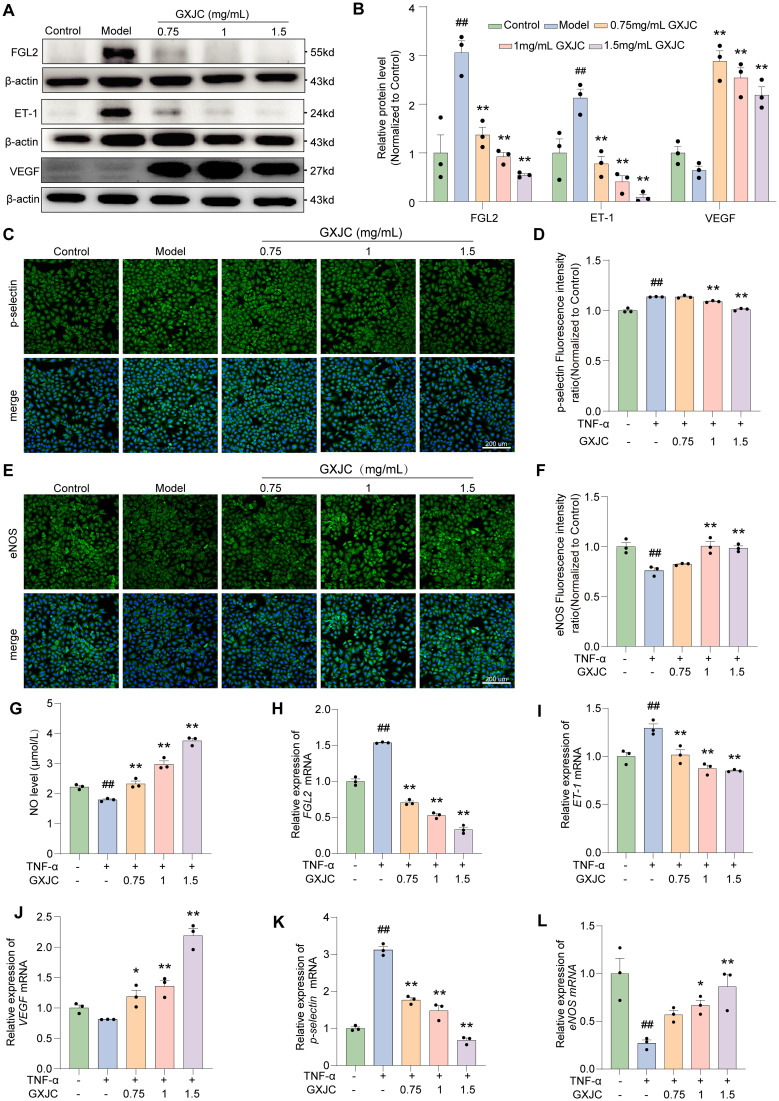
GXJC alleviated TNF-α-induced endothelial cell dysfunction in HUVECs. **(A, B)** The expression of FGL2, ET-1, VEGF were detected by WB after TNF-α stimulation, (n = 3). **(C, D)** Representative immunofluorescence images of p-selectin in HUVECs and quantitative analysis. Scale bar =200 μm (n = 3). **(E, F)** Representative immunofluorescence images of eNOS in HUVECs and quantitative analysis. Scale bar = 200 μm (n = 3). **(G)** Determination of NO Content in Culture Medium Supernatant by Griess method (n = 3). **(H–L)** Relative mRNA expression levels of *FGL2*, *ET-1*, *VEGF*, *p-selectin* and *eNOS* were measured by RT-qPCR in HUVECs (n = 3). ^#^*P* < 0.05, ^##^*P* < 0.01 vs. Control group. ^*^P < 0.05, ^**^P < 0.01 vs. Model group.

These results indicate that GXJC attenuates TNF-α-induced inflammation in HUVECs. To further investigate whether GXJC ameliorates endothelial dysfunction through anti-inflammatory mechanisms, we examined key molecules involved in vascular homeostasis, vasomotor regulation, and endothelial activation. Western blotting analysis revealed that compared to the Control group, TNF-α stimulation significantly increased the protein expression of FGL2 and ET-1, whereas VEGF expression showed a decreasing trend without statistical significance. Notably, GXJC treatment significantly reduced FGL2 and ET-1 protein levels and markedly increased VEGF expression compared with the Model group (**Figures 6A, B**). Immunofluorescence staining further revealed that TNF-α stimulation significantly upregulated p-selectin expression and downregulated eNOS expression in HUVECs compared to Control, effects which were reversed by GXJC administration (**Figures 6C–F**). Concurrently, NO production in HUVECs, measured using the Griess assay, was significantly reduced following TNF-α modeling but increased by GXJC treatment (**Figure 6G**). In agreement with the protein-level findings, RT-qPCR analysis showed that GXJC significantly decreased TNF-α-induced mRNA expression of FGL2, ET-1, and P-selectin, while increasing the mRNA expression of VEGF and eNOS (**Figures 6H–L**). Collectively, these *in vitro* findings indicate that GXJC ameliorates endothelial dysfunction at the cellular level through dual mechanisms: firstly, by restoring the eNOS/NO axis and upregulating VEGF to enhance the vasodilatory and reparative potential of endothelial cells; secondly, by suppressing the FGL2/ET-1 pathway to attenuate pro-thrombotic and vasoconstrictive signaling. These molecular effects act synergistically, ultimately alleviating inflammation-induced cellular dysfunction and exerting direct endothelial protective effects.

Building upon the aforementioned findings, the active compounds and endothelial function-related targets of GXJC were identified. A compound-target network was constructed for the key active ingredients and subjected to topological analysis. As illustrated in **Supplementary Figure S3**, the top 10 compounds with the highest degree values were selected as core candidates. Notably, dihydroisotanshinone I (Compound 96) exhibited the highest node degree, suggesting a pivotal role in the multi-target therapeutic mechanism of GXJC.

Subsequently, molecular docking simulations were performed to evaluate the binding affinities between these top 10 compounds and three critical targets: TNF, eNOS, and ET-1 (**Supplementary Table 4**). Dihydroisotanshinone I demonstrated highly favorable binding affinities across all three targets, with predicted binding energies of -11.4, -15.0, and -9.2 kcal/mol for TNF, eNOS, and ET-1, respectively. Other compounds, including scutellarein, kaempferol, and cianidanol, also exhibited robust binding activities, particularly toward eNOS and TNF, with binding energies consistently below -8.0 kcal/mol.

To further elucidate the binding modes of the most promising interactions, ligand-target pairs with binding energies ≤ -9.0 kcal/mol were selected for 3D structural visualization. As depicted in **Supplementary Figure S4**, stable binding conformations were observed between the selected compounds and their respective targets. Specifically, strong interactions were visualized for the following pairs: scutellarein-TNF (-9.0 kcal/mol, **Supplementary Figure S4A**), cianidanol-TNF (-9.1 kcal/mol, **Supplementary Figure S4B**), dihydroisotanshinone I-TNF (-11.4 kcal/mol, **Supplementary Figure S4C**), scutellarein-eNOS (-9.1 kcal/mol, **Supplementary Figure S4D**), kaempferol-eNOS (-9.3 kcal/mol, **Supplementary Figure S4E**), dihydroisotanshinone I-eNOS (-15.0 kcal/mol, **Supplementary Figure S4F**), and dihydroisotanshinone I-ET-1 (-9.2 kcal/mol, **Supplementary Figure S4G**). Collectively, these findings suggest that dihydroisotanshinone I, scutellarein, kaempferol, and cianidanol are the primary bioactive components mediating the pharmacological effects of GXJC.

## 4 Discussion

**Figure 7:**
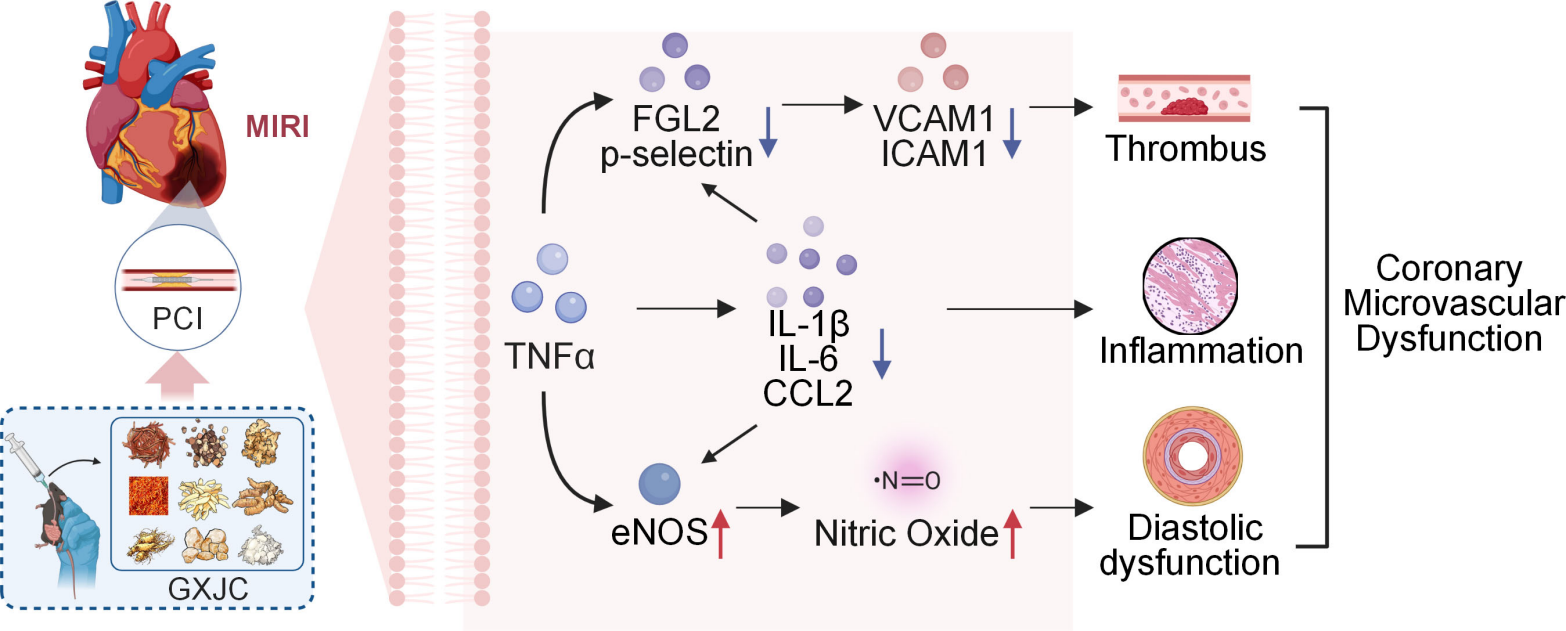
The potential mechanism of GXJC in treating MIRI-induced CMD.

In this study, 256 major chemical components were identified from the GXJC extract using UHPLC-Q-Exactive HRMS. To generate initial hypotheses regarding its therapeutic mechanisms, network pharmacology was employed, predicting 429 potential targets for MIRI treatment, including 30 core targets screened by PPI network topology. GO and KEGG analyses suggested that these targets are mainly involved in hypoxia inhibition, anti-inflammatory and anti-apoptotic processes, particularly inflammatory mediator regulation of TRP channels, TNF signaling pathways, and apoptosis pathways. Subsequent *in vivo* and *in vitro* experiments were designed to investigate specific predicted pathways (such as the TNF signaling pathway) rather than to validate the entire predicted network. These empirical results demonstrated that GXJC alleviated MIRI-induced myocardial injury and associated microvascular dysfunction by improving endothelial dysfunction and microcirculatory impairment. GXJC reduced infarct size and apoptosis, preserved vascular structural integrity and ameliorated MIRI-induced endothelial injury, as evidenced by increased CD31 and α-SMA expression, suppressed inflammatory mediators, downregulated ET-1, FGL2, and P-selectin, and enhanced eNOS expression and NO production. In HUVECs, GXJC restored cellular morphology and attenuated inflammation and endothelial dysfunction. Overall, our findings confirm that GXJC attenuates MIRI-induced myocardial injury and associated microvascular dysfunction by inhibiting vascular inflammation, regulating the eNOS/NO/ET-1 axis, and reducing FGL2/P-selectin-mediated endothelial injury and thrombosis (**Figure 7**).

Endothelial dysfunction constitutes a key feature underlying CMD (32), and is primarily characterized by impaired vasomotor function (33). Endothelial cells regulate vascular relaxation mainly through the production of NO, which is controlled by eNOS expression and activity (34). NO is recognized as a principal mediator of vasodilation and cardiovascular homeostasis (35). In contrast, ET-1 is a potent endogenous vasoconstrictor, and vascular contraction is largely mediated by ET-1 signaling (36). Excessive ET-1 signaling promotes vasoconstriction, inflammation, vascular remodeling, and reduced NO bioavailability, thereby exacerbating endothelial dysfunction (37, 38). In this study, CMD mice exhibited abnormal vasomotor regulation, as reflected by decreased eNOS expression and NO production together with elevated ET-1 levels, indicating impaired coronary endothelial function. Previous studies support this mechanism. For example, fasudil preserves endothelial function by inhibiting ROCK signaling, reducing ET-1 and ETA receptor expression, and enhancing NO production (39). Similarly, Tongmai Yangxin pill activates cAMP/PKA and NO/cGMP pathways, upregulating eNOS activity and NO bioavailability to induce coronary microvascular relaxation, thereby alleviating myocardial no-reflow following ischemia-reperfusion (40). Consistent with these findings, our results showed that GXJC increased eNOS expression and NO generation while suppressing ET-1, thereby restoring endothelium-dependent vasomotor function. However, the precise upstream molecular mechanisms responsible for this regulation require further investigation.

CMD is also frequently accompanied by chronic low-grade vascular inflammation (41) and endothelial function is closely linked to inflammatory responses (42). Pro-inflammatory cytokines such as IL-1β, TNF-α, and IL-6, can activate endothelial cells and induce endothelial dysfunction (43). Specifically, TNF-α potentiates vascular inflammation and endothelial impairment by activating NF-κB to stimulate ROS production and disrupt the ET-1/NO balance in arterioles (44). IL-1β induces NOS uncoupling, and promotes the expression of adhesion molecules, such as VCAM-1 and ICAM-1, as well as inflammatory cytokines, including IL-1β and IL-6 (45, 46). CCL2 is a chemokine regulating monocyte/macrophage migration and infiltration, plays a pivotal role in vascular endothelial inflammation (47). In addition, IL-6 trans-signaling further exacerbates inflammatory responses by mediating CCL2 expression and release in human vascular endothelial cells via JAK/STAT3 and PI3K/AKT pathways (48). Conversely, the anti-inflammatory cytokine IL-10 prevents TNF-α-induced endothelium-dependent vasodilation by protecting eNOS expression and augmenting NO bioavailability (49). In our CMD model, the increased levels of IL-1β, IL-6, CCL2, VCAM-1, and ICAM-1, together with decreased IL-10 expression, suggest that MIRI induces cardiac inflammation, upregulates adhesion molecules, disrupts ET-1/NO equilibrium, and culminates in CMD pathogenesis. These findings are consistent with previous reports showing that anthocyanins and their metabolites prevent endothelial dysfunction and inflammation by inhibiting ICAM-1, VCAM-1, IL-6, and CCL2 expression (50). Similarly, key constituents of *Salvia miltiorrhiza*, including cryptotanshinone, tanshinones, and salvianolic acids, have been reported to reduce IL-6, IL-1β, and TNF-α expression, thereby suppressing inflammation, abnormal angiogenesis, and endothelial barrier dysfunction (51). Importantly, our findings reveal that GXJC treatment suppresses IL-6, IL-1β, CCL2, VCAM-1, and ICAM-1 while enhancing IL-10 expression, demonstrating its anti-inflammatory efficacy against CMD.

Substantial evidence indicates that thrombotic and inflammatory mechanisms are closely interconnected and jointly contribute to the progression of microcirculatory impairment (52). Pro-inflammatory cytokines, such as IL-1β, IL-6, and TNF-α, promote platelet activation (53), while activated platelets further modulate innate immune responses and enhance leukocyte recruitment (54). These leukocytes adhere to the vascular endothelium through surface adhesion molecules, including ICAM-1, VCAM-1, and P-selectin, thereby promoting cellular adhesion and inflammatory thrombosis (52). FGL2, which is predominantly expressed in endothelial cells, can directly cleave prothrombin into thrombin and subsequently catalyze the conversion of fibrinogen into fibrin. Through this process, FGL2 promotes *in situ* thrombosis, vascular remodeling, and endothelial apoptosis (55). Previous studies have shown that JAK inhibitors attenuate endothelial prothrombotic activation by suppressing JAK-STAT-mediated P-selectin and IL-6 secretion (56). In addition, reduced FGL2 expression has been reported to improve coronary microvascular function (57). In agreement with these findings, our experimental results showed that GXJC significantly reversed CMD-induced increases in FGL2 and P-selectin expression at both the protein and mRNA levels, suggesting that GXJC may alleviate microvascular thrombosis and endothelial injury by inhibiting FGL2/P-selectin-mediated prothrombotic activation.

Nevertheless, this study has several limitations. First, the lack of pharmacokinetic data limits the extrapolation of *in vitro* GXJC concentrations to *in vivo* exposure. Since crude extracts may not reflect the actual bioactive compounds reaching the endothelium, future serum-pharmacological studies are needed to identify the true *in vivo* active components. Second, while GXJC appears to protect endothelial function via the TNF pathway, the precise downstream mechanisms remain unclear. Third, microcirculatory improvements were inferred mainly from molecular and histological markers, lacking direct hemodynamic assessments such as coronary flow reserve or myocardial perfusion imaging. Finally, because these findings are restricted to preclinical models, clinical trials are essential to confirm the efficacy and safety of GXJC in patients with CMD.

## 5 Conclusion

In conclusion, GXJC attenuates inflammatory responses and ameliorates microvascular endothelial injury following MIRI. Furthermore, it restores microvascular endothelial homeostasis by preserving microvascular structural integrity, inhibiting endothelial apoptosis, and modulating endothelial activation. This study provides mechanistic evidence for the pharmacological effects of GXJC against MIRI-induced myocardial injury and associated microvascular endothelial dysfunction, offering an experimental basis for its potential clinical application in microvascular complications post-revascularization.

## Data Availability

The original contributions presented in the study are included in the article/**Supplementary Material**. Further inquiries can be directed to the corresponding author.
