# Supplementary material for: Guanxinjing ameliorates coronary microvascular dysfunction in myocardial ischemia-reperfusion injury by alleviating inflammation and restoring endothelial function
Source: Front Immunol. 2026 Jul 17;17:1846953. doi: 10.3389/fimmu.2026.1846953 (PMC13423699; doi:10.3389/fimmu.2026.1846953)
Supplement: Supplementary file 1 [file Supplementaryfile1.docx]

**Supplementary Materials**


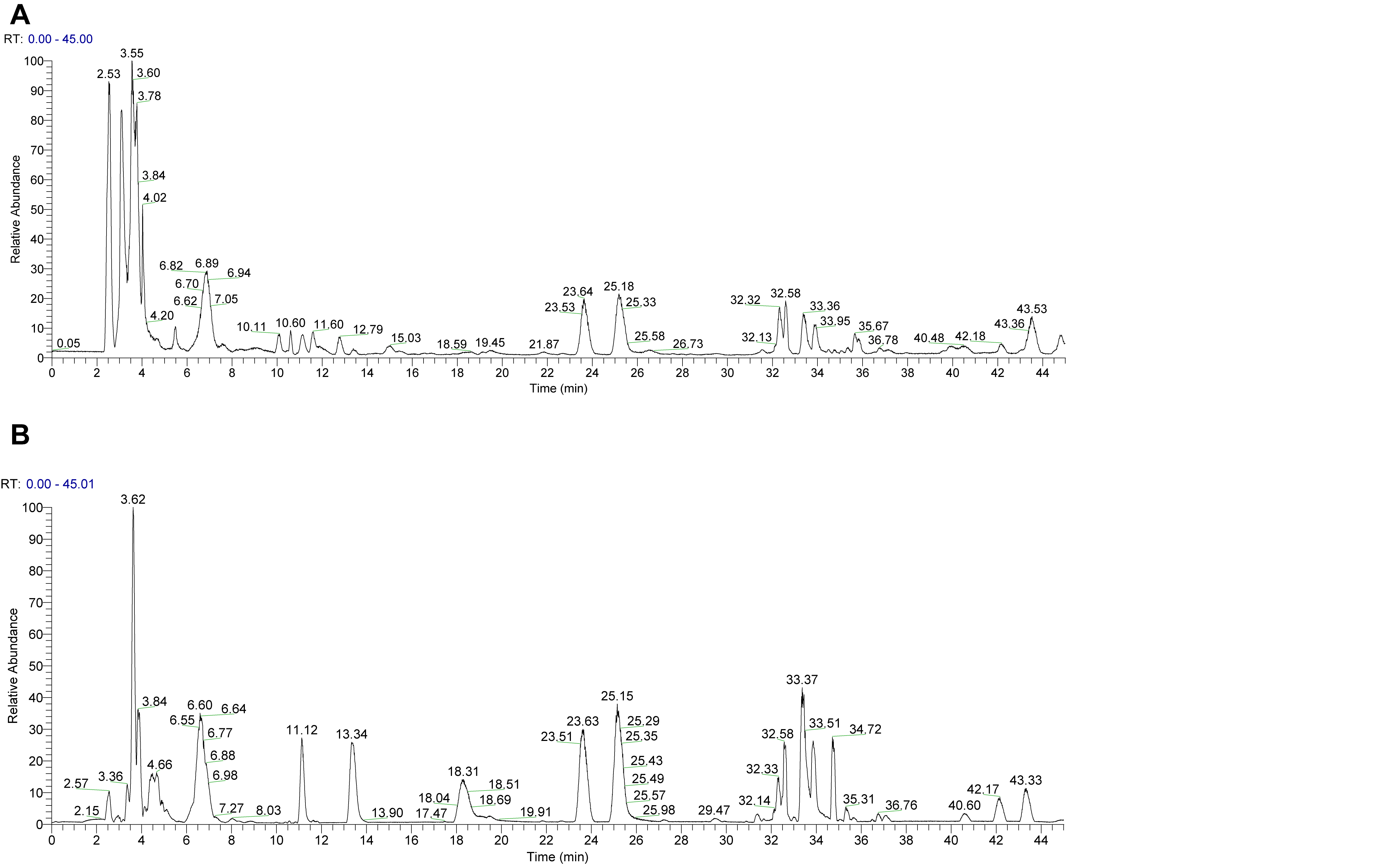


**Fig. S1** The chemical components of GXJC were analyzed using UHPLC-Q-Exactive HRMS. **A** Base peak chromatogram in positive ionization mode. **B** Base peak chromatogram in negative ionization mode.


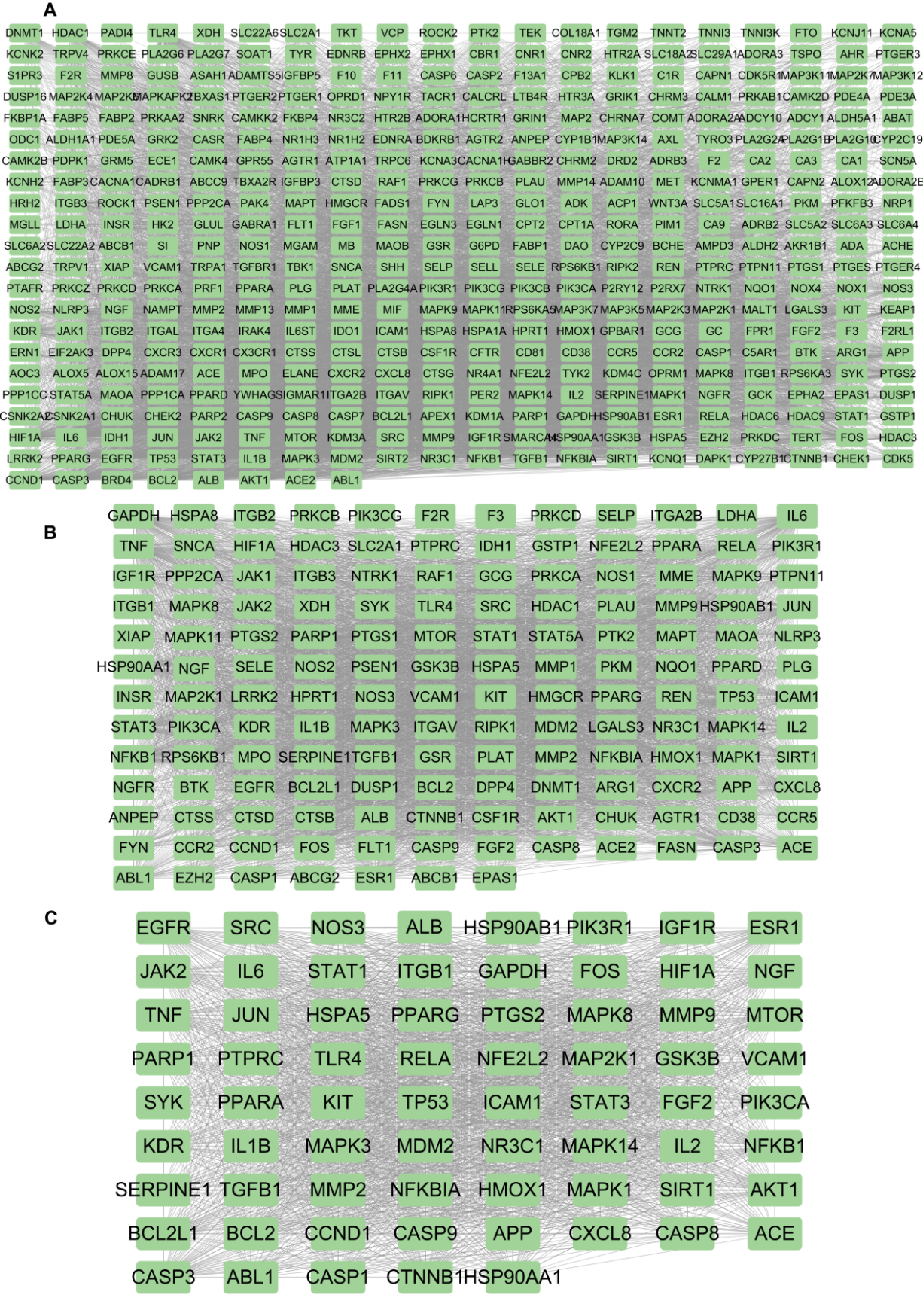
**Fig. S2** Identification and Screening of Potential Therapeutic Targets of GXJC for MIRI-Induced CMD. **A** The visualized original PPI network of the intersecting targets. **B** The PPI networks after one rounds of screening based on topological algorithms (DC, CC, BC, NC, EC, LAC). **C** The PPI networks after two rounds of screening based on topological algorithms.


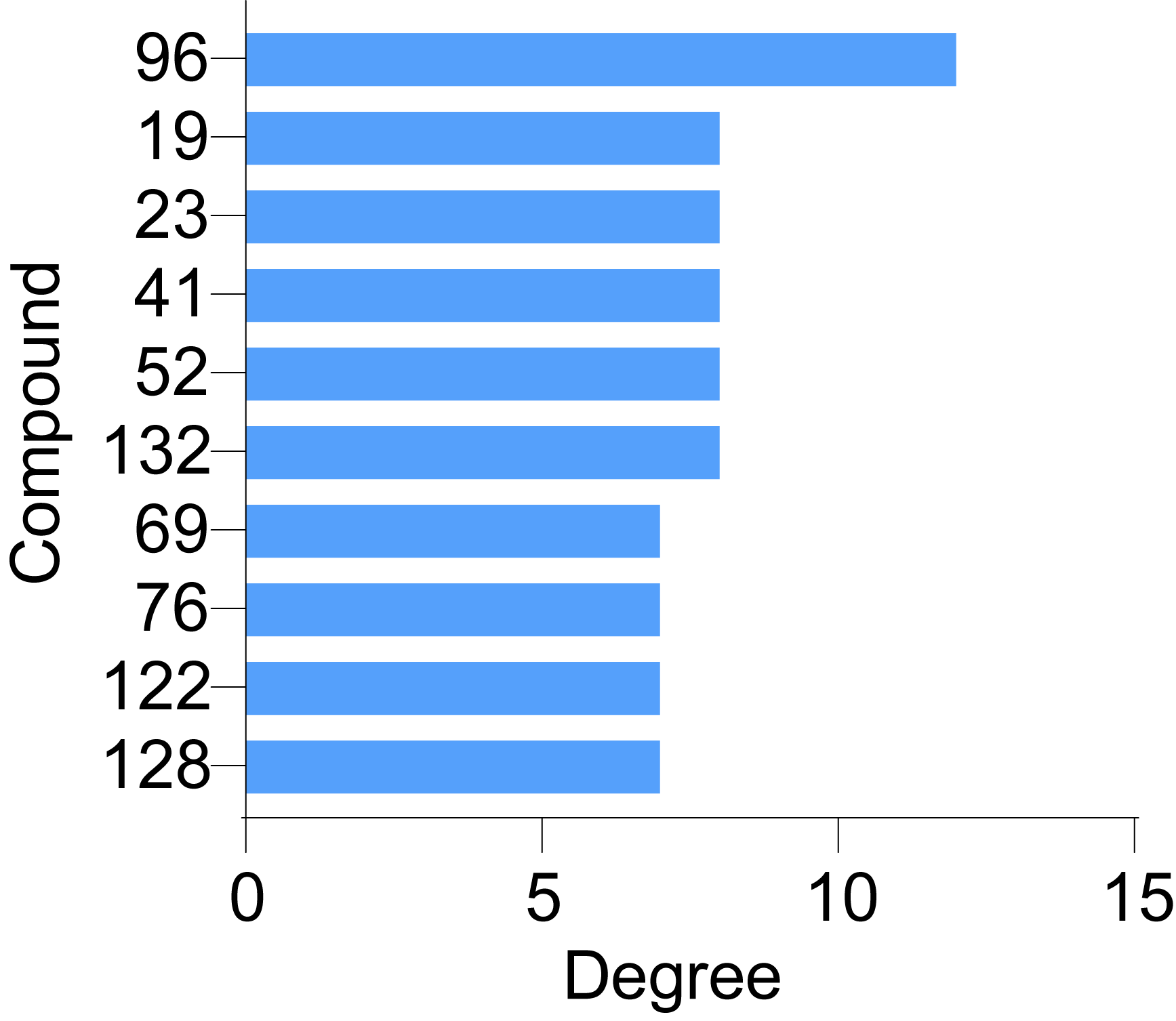


**Fig. S3** Top 10 compounds of GXJC based on degree values in the compound-target network.


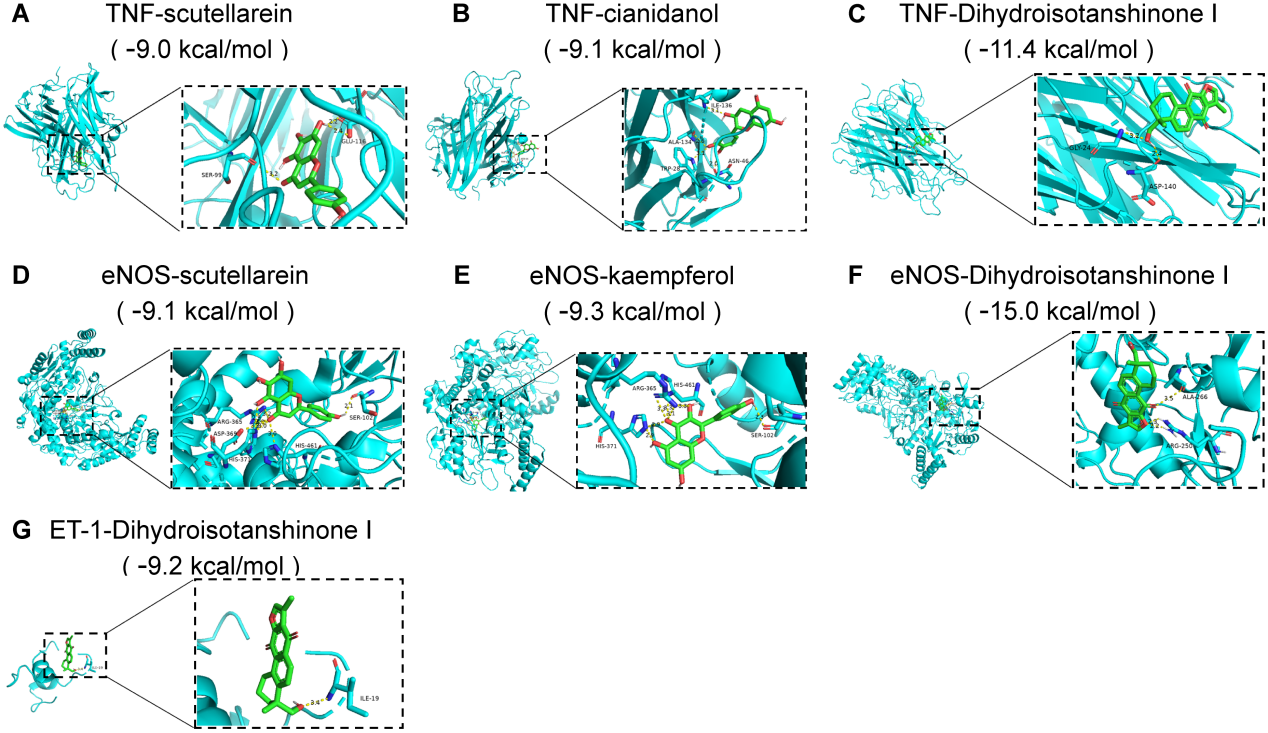


**Fig S4.** Molecular docking results of GXJC-derived active components with key targets (TNF, eNOS, and ET-1). Only ligand-target pairs with binding energies lower than -9.0 kcal/mol are presented. The calculated binding affinities are as follows: (A) scutellarein-TNF (-9.0 kcal/mol); (B) cianidanol-TNF (-9.1 kcal/mol); (C) dihydroisotanshinone I-TNF (-11.4 kcal/mol); (D) scutellarein-eNOS (-9.1 kcal/mol); (E) kaempferol-eNOS (-9.3 kcal/mol); (F) dihydroisotanshinone I-eNOS (-15.0 kcal/mol); (G) dihydroisotanshinone I-ET-1 (-9.2 kcal/mol). Hydrogen bonds and hydrophobic interactions are depicted as dashed lines.

**Supplementary Table S1.** Primer sequences for qRT-PCR.

| Genes | Forward Primer | Reverse Primer |
| --- | --- | --- |
| hIL-6 | TTCGGTCCAGTTGCCTTCTC | TCACCAGGCAAGTCTCCTCA |
| hIL10 | ATGCACAGCTCAGCACTGC | TCAGTTTCGTATCTTCATTGTC |
| hIL-1β | TGATGGCTTATTACAGTGGCAATG | GTAGTGGTGGTCGGAGATTCG |
| hCCL2 | GCCTTAAGTAATGTTAATTCTTAT | GGTGTAATAGTTACAAAATATTCA |
| hICAM-1 | TATGGCAACGACTCCTTCT | CATTCAGCGTCACCTTGG |
| hVCAM-1 | TCTCATTGACTTGCAGCACCACAG | CCCTCATTCGTCACCTTCCCATTC |
| hVEGF | CCTGGTGGACATCTTCCAGGAGTACC | CTCACCGCCTCGGCTTGTCA |
| hET-1 | TCCTCTGCTGGTTCCTGACT | CAGAAACTCCACCCCTGTGT |
| hP-selectin | TGCTCAGAACTACATGT | AGGACTCGGGTCAAATG |
| hFGL2 | ACTGTGACATGGAGACCATG | TCCTTACTCTTGGTCAGAAG |
| heNOS | GCTGCGCCAGGCTCTCACCTTC | GGCTGCAGCCCTTTGCTCTCAA |
| hβ-actin | AAGGATTCCTATGTGGGCGAC | CGTACAGGGATAGCACAGCC |

**Abbreviations:** hIL-6：Human Interleukin-6; hIL-10: human Interleukin-10; hIL-1β: human Interleukin-1 beta; hCCL2: human C-C Motif Chemokine Ligand 2; hICAM-1: human Intercellular Adhesion Molecule 1; hVCAM: human Vascular Cell Adhesion Molecule 1; hVEGF: human Vascular Endothelial Growth Factor; hET-1: human Endothelin-1; hFGL2: human Fibrinogen-like Protein 2; heNOS: human Endothelial Nitric Oxide Synthase.

**Supplementary Table S2.** Identification of compounds by UHPLC-HRMS.

| **NO.** | **Name** | **RT [min]** | **Formula** | **CAS No.** | **Molecular Weight** | **Ion Type** | **Molecular Ion (m/z)** | **Error (ppm)** | **Main Product Ion (m/z)** |
| --- | --- | --- | --- | --- | --- | --- | --- | --- | --- |
|  |  |  |  |  |  |  |  |  |  |
| 1 | L-histidine | 2.97 | C₆H₉N₃O₂ | 71-00-1 | 155.06948 | [M+H]+ | 156.07635 | -2.44 | 130.06261,110.07126,95.06029 |
| 2 | D-serine | 3.277 | C₃H₇NO₃ | 312-84-5 | 105.04259 | [M+H]+ | 106.04993 | 0.54 | 90.60011,86.06035,60.08139 |
| 3 | L-threonine | 3.345 | C₄H₉NO₃ | 6028-28-0 | 119.05824 | [M+H]+ | 120.06541 | -0.95 | 102.05508,74.06049,56.05014 |
| 4 | fructose | 3.368 | C₆H₁₂O₆ | 57-48-7 | 180.06339 | [M+H]+ | 181.07013 | -2.97 | 162.07547,109.02842,81.03389 |
|  |  |  |  |  |  | [M-H]- | 179.05522 | -4.56 | 89.02316,71.01260,59.01260 |
| 5 | daphnoretin | 3.369 | C₁₉H₁₂O₆ | 2034-69-7 | 336.06339 | [M+H]+ | 337.07083 | 0.5 | 338.17496,175.11850,70.06564 |
| 6 | L-glutamic acid | 3.413 | C₅H₉NO₄ | 56-86-0 | 147.05316 | [M+H]+ | 148.05998 | -3.07 | 102.05508,74.06049,56.05014 |
| 7 | raffinose | 3.52 | C₁₈H₃₂O₁₆ | 512-69-6 | 504.16903 | [M+H]+ | 505.17374 | -4.89 | 324.12851,306.11652,163.05959 |
|  |  |  |  |  |  | [M-H]- | 503.16156 | -0.46 | 383.11972,179.05545,89.02319 |
| 8 | mannitol | 3.545 | C₆H₁₄O₆ | 87-78-5 | 182.07904 | [M+H]+ | 183.08577 | -2.99 | 129.05429,104.10711,69.03404 |
|  |  |  |  |  |  | [M-H]- | 181.07086 | -4.81 | 163.06029,101.02324,89.02322 |
| 9 | dimethyl succinate | 3.549 | C₆H₁₀O₄ | 106-65-0 | 146.05791 | [M+H]+ | 147.06467 | -3.55 | 130.04956,102.05505,84004472 |
| 10 | oxypeucedanin | 3.551 | C₁₆H₁₄O₅ | 737-52-0 | 286.08412 | [M+H]+ | 287.09195 | 1.92 | 200.04385,126.05476,108.04442 |
|  |  |  |  |  |  | [M-H]- | 285.07697 | 0.41 | 241.08690,214.06322,147.04443 |
| 11 | verbascose | 3.557 | C₃₀H₅₂O₂₆ | 546-62-3 | 828.27468 | [M+H]+ | 829.27808 | -4.69 | 762.79248,325.11139,163.05956 |
|  |  |  |  |  |  | [M-H]- | 827.26575 | -2 | 383.11996,179.05554,89.02321 |
| 12 | 5-hydroxymethylfurfural | 3.592 | C₆H₆O₃ | 67-47-0 | 126.03169 | [M+H]+ | 127.03873 | -1.87 | 109.02844,99.04417,81.03386 |
| 13 | salvianic acid A | 3.598 | C₉H₁₀O₅ | 76822-21-4 | 198.05282 | [M+H]+ | 199.05963 | -2.37 | 182.11679,156.06493,97.02865 |
| 14 | 4-hydroxy-3,5-dimethoxycinnamic acid | 3.612 | C₁₁H₁₂O₅ | 530-59-6 | 224.06847 | [M+H]+ | 225.07494 | -3.63 | / |
|  |  |  |  |  |  | [M-H]- | 223.06076 | -1.95 | / |
| 15 | L-valine | 3.656 | C₅H₁₁NO₂ | 72-18-4 | 117.07898 | [M+H]+ | 118.08609 | -1.41 | 85.53379,70.06566,59.07632 |
| 16 | nystose | 3.665 | C₂₄H₄₂O₂₁ | 13133-07-8 | 666.22186 | [M+H]+ | 667.2262 | -4.41 | 552.08493,325.11130,163.05959 |
|  |  |  |  |  |  | [M-H]- | 665.21368 | -0.66 | 485.15152,383.11984,179.05547 |
| 17 | ribonolactone | 3.697 | C₅H₈O₅ | 533-8-6 | 148.03717 | [M+H]+ | 149.04399 | -3.1 | 130.09723,84.04474,70.06567 |
| 18 | L-proline | 3.732 | C₅H₉NO₂ | 147-85-3 | 115.06333 | [M+H]+ | 116.07056 | -0.42 | 91.61898,70.06564 |
| 19 | pyrogallol | 3.861 | C₆H₆O₃ | 87-66-1 | 126.03169 | [M+H]+ | 127.03873 | -1.87 | 108.04440,81.03384,69.03401 |
| 20 | soyasapogenol A | 3.875 | C₁₂H₂₂O₁₁ | 20880-64-2 | 342.11621 | [M+H]+ | 343.12201 | -4.31 | 306.11758,145.04915,127.03875 |
|  |  |  |  |  |  | [M-H]- | 341.10858 | -0.67 | 179.05522,119.03386,89.02318 |
| 21 | glucuronic acid | 3.905 | C₆H₁₀O₇ | 6556/12/3 | 194.04265 | [M-H]- | 193.03456 | -4.2 | 127.03901,103.00260,85.02824 |
| 22 | vitamin C | 3.957 | C₆H₈O₆ | 50-81-7 | 176.03209 | [M+H]+ | 177.03871 | -4.26 | 130.08603,112.07567,87.04436 |
|  |  |  |  |  |  | [M-H]- | 175.02414 | -3.82 | 157.01340,146.96031,118.96503 |
| 23 | dimethyl lithospermate B | 4.007 | C₃₈H₃₄O₁₆ | 875313-64-7 | 746.18468 | [M+H]+ | 747.19281 | 1.14 | / |
|  |  |  |  |  |  | [M-H]- | 745.18015 | 3.14 | / |
| 24 | adenine | 4.035 | C₅H₅N₅ | 73-24-5 | 135.0545 | [M+H]+ | 136.06151 | -1.95 | 119.03490,94.04026,79.05462 |
| 25 | L-2-amino-3-(5-hydroxyindolyl)propionic acid | 4.048 | C₁₁H₁₂N₂O₃ | 314062-44-7 | 220.08479 | [M+H]+ | 221.0914 | -3.04 | / |
| 26 | 2-acetylpyrrole | 4.079 | C₆H₇NO | 1072-83-9 | 109.05276 | [M+H]+ | 110.06013 | 0.8 | 119.03490,94.04026,79.05462 |
| 27 | methyl pyruvate | 4.909 | C₄H₆O₃ | 600-22-6 | 102.03169 | [M+H]+ | 103.03922 | 2.4 | 8502872,74.06055,60.08140 |
| 28 | succinamic acid | 4.923 | C₄H₇NO₃ | 638-32-4 | 117.04259 | [M+H]+ | 118.0499 | 0.29 | 98.98433,72.08128,70.06565 |
| 29 | 1-kestose | 5.441 | C₁₉H₃₄O₁₆ | 470-69-9 | 518.18468 | [M+H]+ | 519.19031 | -4.06 | 409.13010,277.08884 |
|  |  |  |  |  |  | [M-H]- | 517.17786 | 0.99 | 385.13605,253.09300,89.02321 |
| 30 | stachyose | 5.477 | C₂₄H₄₂O₂₁ | 470-55-3 | 666.22186 | [M-H]- | 665.21368 | -0.7 | 545.17542,179.05545,89.02316 |
| 31 | 2-hydroxy-2-isopropylsuccinic acid | 5.555 | C₇H₁₂O₅ | 3237-44-3 | 176.06847 | [M+H]+ | 177.07564 | -0.64 | 146.96085,130.08600,84.08112 |
| 32 | nicotinic acid | 5.613 | C₆H₅NO₂ | 59-67-6 | 123.03203 | [M+H]+ | 124.03916 | -1.16 | 105.03716,95.04946,79.05466 |
| 33 | trans-aconitic acid | 6.636 | C₆H₆O₆ | 4023-65-8 | 174.01644 | [M+H]+ | 175.02313 | -3.35 | 157.01234,139.00223,129.01793 |
| 34 | citric acid | 6.651 | C₆H₈O₇ | 77-92-9 | 192.027 | [M+H]+ | 193.03363 | -3.38 | 157.01268,139.00209,129.01797 |
|  |  |  |  |  |  | [M-H]- | 191.01898 | -3.87 | 111.00771,87.00757 |
| 35 | N-acetyl-2-oxopropanamide | 6.862 | C₅H₇NO₃ | 70881-55-9 | 129.04259 | [M+H]+ | 130.04953 | -2.63 | 111.20065,84.04469 |
| 36 | L-leucine | 6.97 | C₆H₁₃NO₂ | 61-90-5 | 131.09463 | [M+H]+ | 132.10139 | -3.89 | 86.09671,69.07038 |
| 37 | protocatechuic acid | 7.039 | C₇H₆O₄ | 99-50-3 | 154.02661 | [M+H]+ | 155.03336 | -3.44 | 139.00215,125.00222,111.00770 |
| 38 | p-Hydroxybenzaldehyde | 7.569 | C₇H₆O₂ | 123-08-0 | 122.03678 | [M+H]+ | 123.04372 | -2.72 | 112.03919,95.04926,79.05452 |
| 39 | tyrosine | 7.578 | C₉H₁₁NO₃ | 60-18-4 | 181.07389 | [M+H]+ | 182.0808 | -2.09 | 165.05402,136.07524,123.04377 |
|  |  |  |  |  |  | [M-H]- | 180.06601 | -3.35 | 163.03929,119.04910,112.98465 |
| 40 | salvianolic acid F | 7.794 | C₁₇H₁₄O₆ | 158732-59-3 | 314.07904 | [M+H]+ | 315.086 | -1.01 | 296.07297,224.05182,152.03175 |
|  |  |  |  |  |  | [M-H]- | 313.07208 | 1.02 | 269.08221,254.05855,109.02843 |
| 41 | succinic acid | 8.108 | C₄H₆O₄ | 110-15-6 | 118.02661 | [M+H]+ | 119.03381 | -0.61 | 101.02345,90.94790,72.08123 |
| 42 | uridine | 8.12 | C₉H₁₂N₂O₆ | 58-96-8 | 244.06954 | [M+H]+ | 245.07611 | -3.38 | 181.98927,113.03447 |
|  |  |  |  |  |  | [M-H]- | 243.06226 | -0.01 | 200.05592,152.03442,110.02365 |
| 43 | uracil | 8.138 | C₄H₄N₂O₂ | 66-22-8 | 112.02728 | [M+H]+ | 113.0346 | 0.4 | 96.00815,90.94789,72.93756 |
| 44 | 8-debenzoylpaeoniflorin | 9.054 | C₁₆H₂₄O₁₀ | 23532-11-8 | 376.13695 | [M+H]+ | 377.14252 | -4.56 | 237.07240,219.06233,175.07253 |
|  |  |  |  |  |  | [M-H]- | 375.12982 | 1.22 | 345.11935,195.06627,151.07590 |
| 45 | cumalic acid | 9.926 | C₆H₄O₄ | 500-05-0 | 140.01096 | [M+H]+ | 141.01799 | -1.75 | 113.96365,90.94792,72.93758 |
| 46 | methyl 2-O-α-L-fucopyranosyl-β-D-galactoside | 10.081 | C₁₃H₂₄O₁₀ | 24656-23-3 | 340.13695 | [M+H]+ | 341.14423 | 0.01 | / |
|  |  |  |  |  |  | [M-H]- | 339.12994 | 1.29 | 249.12529,207.08708,101.02313 |
| 47 | adenosine | 10.085 | C₁₀H₁₃N₅O₄ | 58-61-7 | 267.09675 | [M+H]+ | 268.10306 | -3.64 | 182.01541,136.06142,115.03958 |
|  |  |  |  |  |  | [M-H]- | 266.08972 | 1.38 | 177.06659,134.04626 |
| 48 | guanosine | 10.598 | C₁₀H₁₃N₅O₅ | 118-00-3 | 283.09167 | [M+H]+ | 284.09787 | -3.83 | 165.12817,206.08020,152.05612 |
|  |  |  |  |  |  | [M-H]- | 282.08463 | 0.82 | 150.04128,133.01465,94.02865 |
| 49 | tyramine | 10.72 | C₈H₁₁NO | 51-67-2 | 137.08406 | [M+H]+ | 138.09111 | -1.68 | 120.04736,91.05443 |
| 50 | shanzhiside methyl ester | 11.112 | C₁₇H₂₆O₁₁ | 64421-28-9 | 406.14751 | [M-H]- | 405.14026 | 0.06 | 359.13498,197.08139,179.07063 |
| 51 | gallic acid | 11.178 | C₇H₆O₅ | 149-91-7 | 170.02152 | [M+H]+ | 171.02831 | -2.91 | 153.01768,127.03868,109.02838 |
| 52 | 1-galloyl-glucose | 11.391 | C₁₃H₁₆O₁₀ | 13405-60-2 | 332.07435 | [M-H]- | 331.06747 | 1.22 | 271.04611,211.02428,169.01347 |
| 53 | L-phenylalanine | 11.587 | C₉H₁₁NO₂ | 63-91-2 | 165.07898 | [M+H]+ | 166.08569 | -3.4 | 165.12817,206.08020,152.05612 |
|  |  |  |  |  |  | [M-H]- | 164.07089 | -4.9 | 147.04425,96.96837,72.00780 |
| 54 | 4,5-dihydroxy-3-methoxybenzoic acid | 11.674 | C₈H₈O₅ | 3934-84-7 | 184.03717 | [M+H]+ | 185.04391 | -2.91 | 167.03339,140.07019,117.97972 |
|  |  |  |  |  |  | [M-H]- | 183.02925 | -3.51 | 163.03932,135.04419,119.04612 |
| 55 | 4-O-β-D-glucopyranosyloxy-benzoic acid | 11.875 | C₁₃H₁₆O₈ | 15397-25-8 | 300.08452 | [M-H]- | 299.07736 | 0.24 | 137.02348,93.03341 |
| 56 | thymine | 12.058 | C₅H₆N₂O₂ | 65-71-4 | 126.04293 | [M+H]+ | 127.04996 | -1.9 | 110.02364,84.96001 |
| 57 | ethyl gallate | 13.379 | C₉H₁₀O₅ | 831-61-8 | 198.05282 | [M+H]+ | 199.05962 | -2.92 | 154.08577,123.04386,74.09687 |
|  |  |  |  |  |  | [M-H]- | 197.04468 | -4.39 | 179.03433,135.04417,72.99182 |
| 58 | hexenal | 13.612 | C₁₂H₁₆N₂O₃ | 56-29-1 | 236.11609 | [M+H]+ | 237.12259 | -3.3 | 211.02718,175.12253,148.11159 |
| 59 | 3-phenylpropanol | 15.119 | C₉H₁₂O | 122-97-4 | 136.08881 | [M+H]+ | 137.09569 | -2.97 | 109.10115,67.05478 |
| 60 | folinic acid | 15.307 | C₂₀H₂₃N₇O₇ | 1958/5/9 | 473.1659 | [M+H]+ | 474.17105 | -4.48 | / |
| 61 | 3,5-dimethyl-p-anisic acid | 15.438 | C₁₀H₁₂O₃ | 21553-46-8 | 180.07864 | [M+H]+ | 181.08536 | -3.13 | 135.08008,120.08062,93.07008 |
| 62 | paeonilactone A | 15.438 | C₁₀H₁₄O₄ | 98751-79-2 | 198.08921 | [M+H]+ | 199.09572 | -4.08 | 181.08530,135.08006,107.04910 |
|  |  |  |  |  |  | [M-H]- | 197.08131 | -1.98 | 179.03438,135.04428,72.99187 |
| 63 | chlorogenic acid | 15.651 | C₁₆H₁₈O₉ | 327-97-9 | 354.09508 | [M+H]+ | 355.10074 | -4.88 | 163.03842,145.02809 |
|  |  |  |  |  |  | [M-H]- | 353.08783 | 0.07 | 191.05553,161.02370 |
| 64 | 4-hydroxycinnamic acid | 16.023 | C₉H₈O₃ | 4501-31-9 | 164.04734 | [M-H]- | 163.03928 | -4.82 | / |
| 65 | oleuropeic acid | 16.501 | C₁₀H₁₆O₃ | 5027-76-9 | 184.10994 | [M+H]+ | 185.11638 | -4.57 | 163.93939,123.94495,105.93525 |
|  |  |  |  |  |  | [M-H]- | 183.10201 | -3.6 | 139.11194,112.98477,59.01251 |
| 66 | mudanpioside F | 16.607 | C₁₆H₂₄O₈ | 172670-08-5 | 344.14712 | [M+H]+ | 345.15271 | -4.62 | 304.11264,165.09109,109.06483 |
|  |  |  |  |  |  | [M-H]- | 343.14008 | 0.8 | 181.08665,151.07541,109.06463 |
| 67 | vulgarin | 17.205 | C₁₅H₂₀O₄ | 3162-56-9 | 264.13616 | [M+H]+ | 265.14249 | -3.59 | 175.07510,135.08014,72.08122 |
|  |  |  |  |  |  | [M-H]- | 263.12891 | 0.09 | 219.13918,204.11531,139.07542 |
| 68 | paeonoside | 17.406 | C₁₅H₂₀O₈ | 20309-70-0 | 328.11582 | [M-H]- | 327.10858 | 0.12 | 165.05486,146.93823 |
| 69 | oxypaeoniflora | 17.519 | C₂₃H₂₈O₁₂ | 39011-91-1 | 496.15808 | [M+H]+ | 497.16293 | -4.73 | 408.13409,197.08009,121.02821 |
|  |  |  |  |  |  | [M-H]- | 495.1506 |  | 345.12009,195.06554,137.02345 |
| 70 | propyl gallate | 17.971 | C₁₀H₁₂O₅ | 121-79-9 | 212.06847 | [M+H]+ | 213.07494 | -3.84 | 158.02640,140.99997,95.04932 |
|  |  |  |  |  |  |  |  |  |  |
|  |  |  |  |  |  | [M-H]- | 211.06078 | -1.99 | 181.04993,163.03951,148.05550 |
| 71 | 3-hydroxy-5-(trifluoromethyl) benzoic acid | 18.274 | C₈H₅F₃O₃ | 328-69-8 | 206.01908 | [M-H]- | 205.01105 | -3.66 | 160.96683,137.02348 |
| 72 | 4-hydroxybenzoic acid | 18.288 | C₇H₆O₃ | 99-96-7 | 138.03169 | [M+H]+ | 139.03859 | -2.76 | 111.04400,93.03368,74.09683 |
| 73 | 5-methylfurfural | 18.325 | C₆H₆O₂ | 620-02-0 | 110.03678 | [M+H]+ | 111.04423 | 1.56 | 96.00949,87.00432 |
| 74 | tetramethylpyrazine | 18.403 | C₈H₁₂N₂ | 1124-11-4 | 136.10005 | [M+H]+ | 137.10692 | -2.99 | 109.06491,91.05444 |
| 75 | isomaltopaeoniflorin | 18.624 | C₂₉H₃₈O₁₆ | 262350-54-9 | 642.21598 | [M+H]+ | 643.22052 | -4.95 | 319.11737,197.07982,105.03359 |
| 76 | albiflorin | 18.628 | C₂₃H₂₈O₁₁ | 39011-90-0 | 480.16316 | [M+H]+ | 481.16837 | -4.32 | 321.10706,188.06985,146.05951 |
|  |  |  |  |  |  | [M-H]- | 479.15552 | -1.36 | 165.05490,121.02845 |
| 77 | myristicin | 18.669 | C₁₁H₁₂O₃ | 607-91-0 | 192.07864 | [M+H]+ | 193.08525 | -3.49 | 161.05914,133.06447,105.06996 |
|  |  |  |  |  |  | [M-H]- | 191.07065 | -3.75 | 147.08060,106.04107,61.98710 |
| 78 | cianidanol | 18.714 | C₁₅H₁₄O₆ | 154-23-4 | 290.07904 | [M-H]- | 289.07159 | -0.58 | / |
| 79 | salicylic acid | 18.902 | C₇H₆O₃ | 69-72-7 | 138.03169 | [M+H]+ | 139.03859 | -2.76 | 111.04401,74.09686,65.03910 |
| 80 | methyl gallate | 19.344 | C₈H₈O₅ | 99-24-1 | 184.03717 | [M-H]- | 183.02908 | -4.42 | / |
| 81 | betanin | 19.93 | C₂₄H₂₆N₂O₁₃ | 7659-95-2 | 550.14349 | [M-H]- | 549.1347 | -2.74 | / |
| 82 | 3,4-dihydroxyphenethyl glucoside | 20.537 | C₁₄H₂₀O₈ | 76873-99-9 | 316.11582 | [M-H]- | 315.1087 | 0.52 | 178.95679,112.98449 |
| 83 | hydroxysafflor yellow A | 20.999 | C₂₇H₃₂O₁₆ | 78281-02-4 | 612.16903 | [M+H]+ | 613.17334 | -4.85 | 433.11078,235.02271,211.02293 |
|  |  |  |  |  |  | [M-H]- | 611.16187 | 0.18 | 491.11923,325.07211,119.04908 |
| 84 | vanillic acid | 21.522 | C₈H₈O₄ | 121-34-6 | 168.04226 | [M+H]+ | 169.04901 | -3.12 | 149.96085,128.95047,111.04407 |
| 85 | sweroside | 21.974 | C₁₆H₂₂O₉ | 14215-86-2 | 358.12638 | [M-H]- | 357.11902 | -0.24 | 195.06569,151.07643,119.04937 |
| 86 | riboflavin | 22.386 | C₁₇H₂₀N₄O₆ | 83-88-5 | 376.13828 | [M-H]- | 375.13089 | 0.79 | 255.08981,201.01718,102.95562 |
| 87 | syringic acid | 22.504 | C₉H₁₀O₅ | 530-57-4 | 198.05282 | [M+H]+ | 199.0594 | -3.52 | 155.06976,140.04637,95.04944 |
| 88 | 3,5-Dimethoxyacetophenone | 22.564 | C₁₀H₁₂O₃ | 39151-19-4 | 180.07864 | [M+H]+ | 181.08522 | -3.89 | 148.05124,131.97386,113.96356 |
|  |  |  |  |  |  | [M-H]- | 179.07053 | -4.67 | 135.08064,112.98452 |
| 89 | 2-hydroxy-3’,4’- dihydroxyacetophenone | 22.674 | C₈H₈O₄ | 29477-54-1 | 168.04226 | [M+H]+ | 169.04901 | -3.26 | 146.96086,128.95044,88.96876 |
| 90 | 2-hydroxynaringenin 4′-O-glucopyranoside | 22.822 | C₂₁H₂₂O₁₁ | 1616614-47-1 | 450.11621 | [M+H]+ | 451.12109 | -4.97 | 301.06937,235.02249,211.02280 |
|  |  |  |  |  |  | [M-H]- | 449.10904 | 0.23 | 359.07755,329.06702,223.02470 |
| 91 | benzoic acid | 23.413 | C₇H₆O₂ | 65-85-0 | 122.03678 | [M+H]+ | 123.04372 | -2.72 | 95.04930,88.02341,79.01820 |
| 92 | 4-hydroxystyrene | 23.598 | C₈H₈O | 2628-17-3 | 120.05751 | [M+H]+ | 121.06433 | -3.68 | 95.04932,88.02345,56.96544 |
| 93 | mudanpioside E | 23.626 | C₂₄H₃₀O₁₃ | 172705-25-8 | 526.16864 | [M-H]- | 525.16071 | -1.25 | 479.15472,121.02845 |
| 94 | 3-O-p-coumaroylquinic acid | 23.689 | C₁₆H₁₈O₈ | 1899-30-5 | 338.10017 | [M-H]- | 337.09219 | -2.06 | 191.05559,173.04559,93.03338 |
| 95 | tanshinone ⅡB | 23.923 | C₁₉H₁₈O₄ | 17397-93-2 | 310.12051 | [M+H]+ | 311.12634 | -4.65 | / |
| 96 | 6-hydroxykaempferol 3,6-diglucoside | 24.454 | C₂₇H₃₀O₁₇ | 142674-16-6 | 626.1483 | [M+H]+ | 627.15308 | -4.17 | 465.10153,303.04868,85.02873 |
|  |  |  |  |  |  | [M-H]- | 625.14142 | 0.63 | 463.08792,299.02005,271.02518 |
| 97 | hydrocinnamic acid | 25.186 | C₉H₁₀O₂ | 501-52-0 | 150.06808 | [M+H]+ | 151.07463 | -4.83 | 123.08021,105.06993,95.08569 |
| 98 | phenol，4-ethyl- | 25.19 | C₈H₁₀O | 123-07-9 | 122.07316 | [M+H]+ | 123.07999 | -3.73 | 105.07006,95.085668,88.02346 |
| 99 | cinnamyl alcohol | 25.2 | C₉H₁₀O | 104-54-1 | 134.07316 | [M+H]+ | 135.07985 | -4.42 | 117.06978,91.05447,79.05460 |
| 100 | syrigin | 25.861 | C₁₇H₂₄O₉ | 118-34-3 | 372.14203 | [M-H]- | 371.1348 | 0.11 | 322.09725,249.06274,121.02850 |
| 101 | p-coumaroyl-β-d-glucose | 26.192 | C₁₅H₁₈O₈ | 7139-64-2 | 326.10017 | [M-H]- | 325.0932 | 0.95 | 163.03922,119.04918 |
| 102 | isovanillin | 26.344 | C₈H₈O₃ | 621-59-0 | 152.04734 | [M+H]+ | 153.05414 | -3.17 | 123.08022,105.06992,95.08567 |
| 103 | phenylalanine | 26.543 | C₁₀H₁₃NO₂ | 15099-85-1 | 179.09463 | [M+H]+ | 180.10126 | -3.62 | 152.09074,138.09093,112.07565 |
|  |  |  |  |  |  | [M-H]- | 178.08659 | -4.23 | 150.95343,135.04416,71.01264 |
| 104 | ginsenoside F1 | 26.733 | C₃₆H₆₂O₉ | 53963-43-2 | 638.43938 | [M+H]+ | 639.44348 | -4.98 | / |
| 105 | methyl 2-aminobenzoate | 26.846 | C₈H₉NO₂ | 134-20-3 | 151.06333 | [M+H]+ | 152.07011 | -3.25 | 134.05963,125.05940,111.04401 |
| 106 | 2-coumarate | 27.257 | C₉H₈O₃ | 583-17-5 | 164.04734 | [M+H]+ | 165.05412 | -3.03 | 147.04363,119.04893,91.05437 |
|  |  |  |  |  |  |  |  |  |  |
|  |  |  |  |  |  | [M-H]- | 163.03926 | -4.92 | 119.04921,96.96797 |
| 107 | leucoside | 27.394 | C₂₆H₂₈O₁₅ | 27661-51-4 | 580.14282 | [M-H]- | 579.13635 | 1.4 | / |
| 108 | hyperin | 27.399 | C₂₁H₂₀O₁₂ | 482-36-0 | 464.09548 | [M+H]+ | 465.10049 | -4.82 | 303.04852,85.02872 |
|  |  |  |  |  |  |  |  |  |  |
|  |  |  |  |  |  | [M-H]- | 463.08859 | 0.85 | 301.03491,272.03317,165.99022 |
| 109 | dihydrocaffeic acid | 27.851 | C₉H₁₀O₄ | 1078-61-1 | 182.05791 | [M+H]+ | 183.06459 | -3.27 | 159.96855,131.97394,113.96360 |
|  |  |  |  |  |  | [M-H]- | 181.0499 | -4.04 | 149.02359,135.04431,112.98455 |
| 110 | perlolyrine | 28.449 | C₁₆H₁₂N₂O₂ | 29700-20-7 | 264.08988 | [M+H]+ | 265.0961 | -3.99 | 247.08572,206.08319 |
|  |  |  |  |  |  | [M-H]- | 263.08264 | 0.15 | 233.07166,205.07664,167.88910 |
| 111 | rutin | 28.631 | C₂₇H₃₀O₁₆ | 153-18-4 | 610.15338 | [M+H]+ | 611.15759 | -4.93 | 303.04871,85.02877 |
|  |  |  |  |  |  | [M-H]- | 609.14648 | 0.62 | 284.03287,255.02893,121.02855 |
| 112 | N-cis-p-coumaroyloctopamine | 29.109 | C₁₇H₁₇NO₄ | 180050-82-2 | 299.11576 | [M-H]- | 298.10858 | 0.34 | / |
| 113 | tinctormine | 29.151 | C₂₇H₃₁NO₁₄ | 149475-43-4 | 593.17445 | [M-H]- | 592.16766 | 0.82 | 364.08301.339.07480.112.98455 |
| 114 | senkyunolide I | 29.496 | C₁₂H₁₆O₄ | 94596-28-8 | 224.10486 | [M+H]+ | 225.11111 | -4.56 | / |
|  |  |  |  |  |  | [M-H]- | 223.09729 | -1.3 | / |
| 115 | ferulic Acid | 29.53 | C₁₀H₁₀O₄ | 1135-24-6 | 194.05791 | [M+H]+ | 195.06441 | -4.01 | 177.05396,145.02791,117.03329 |
|  |  |  |  |  |  | [M-H]- | 193.04999 | -3.32 | 178.02650,149.05994,134.03638 |
| 116 | isoquercitrin | 29.853 | C₂₁H₂₀O₁₂ | 21637-25-2 | 464.09548 | [M+H]+ | 465.10071 | -4.41 | 303.04871,153.12686,135.11653 |
| 117 | salviaflaside | 30.274 | C₂₄H₂₆O₁₃ | 178895-25-5 | 522.13734 | [M-H]- | 521.13043 | 0.71 | 323.07751,161.02356,112.98452 |
| 118 | neocarthamin | 30.693 | C₂₁H₂₂O₁₁ | 519-94-0 | 450.11621 | [M+H]+ | 451.1217 | -3.93 | 289.06946,169.01257,147.04359 |
|  |  |  |  |  |  | [M-H]- | 449.10938 | 0.98 | 287.05591,166.99774,119.04926 |
| 119 | multiflorin B | 30.893 | C₂₇H₃₀O₁₅ | 52657-01-9 | 594.15847 | [M+H]+ | 595.16309 | -4.39 | 449.10663,287.05377 |
|  |  |  |  |  |  | [M-H]- | 593.15198 | 1.32 | 473.10965,341.06668,153.01836 |
| 120 | scutellarein | 30.904 | C₁₅H₁₀O₆ | 529-53-3 | 286.04774 | [M+H]+ | 287.05386 | -4.02 | 241.04883,204.35501,105.03364 |
| 121 | trijuganone C | 31.041 | C₂₀H₂₀O₅ | 135247-94-8 | 340.13107 | [M+H]+ | 341.13696 | -4.08 | / |
| 122 | isorhamnetin | 31.049 | C₁₆H₁₂O₇ | 480-19-3 | 316.0583 | [M+H]+ | 317.06409 | -4.72 | 209.16408,114.09138 |
|  |  |  |  |  |  | [M-H]- | 315.05118 | 0.48 | 180.97273,112.98454 |
| 123 | 1-hydroxypinoresinol 1-O-glucoside | 31.157 | C₂₆H₃₂O₁₂ | 81495-71-8 | 536.18938 | [M-H]- | 535.18207 | -0.06 | 295.06128,185.02379,109.02836 |
| 124 | ginsenoside Rd | 31.264 | C₄₈H₈₂O₁₉ | 52705-93-8 | 962.54503 | [M+H]+ | 963.547 | -4.93 | / |
| 125 | salvianolic acid D | 31.378 | C₂₀H₁₈O₁₀ | 142998-47-8 | 418.09 | [M-H]- | 417.0827 | -0.04 | 373.09323,197.04500,175.03934 |
| 126 | senkyunolide J | 31.553 | C₁₂H₁₈O₄ | 94530-86-6 | 226.12051 | [M+H]+ | 227.12672 | -4.96 | 209.11649,191.10606,153.05418 |
|  |  |  |  |  |  | [M-H]- | 225.11252 | -3.16 | 181.12332,163.11246,124.05192 |
| 127 | 3-hydroxysenkyunolide A | 31.56 | C₁₂H₁₆O₃ | 94530-85-5 | 208.10994 | [M+H]+ | 209.11624 | -4.71 | 191.10605,163.11121,153.05411 |
| 128 | rhodionin | 31.568 | C₂₁H₂₀O₁₁ | 85571-15-9 | 448.10056 | [M+H]+ | 449.10556 | -4.92 | 287.05383,85.02872 |
|  |  |  |  |  |  | [M-H]- | 447.09326 | -0.05 | 284.03271,255.02988,227.03502 |
| 129 | cartormin | 31.664 | C₂₇H₂₉NO₁₃ | 273917-39-8 | 575.16389 | [M-H]- | 574.15656 | -0.07 | 424.10397,364.08301,244.02499 |
| 130 | cinnamaldehyde | 32.309 | C₉H₈O | 104-55-2 | 132.05751 | [M+H]+ | 133.06424 | -4.18 | 105.06995,103.05434,79.05466 |
| 131 | paeoniflorin | 32.31 | C₂₃H₂₈O₁₁ | 23180-57-6 | 480.16316 | [M+H]+ | 197.07986 | -4.95 | 357.12045,247.32248,121.02840 |
| 132 | paeonilactone B | 32.31 | C₁₀H₁₂O₄ | 98751-78-1 | 196.07356 | [M-H]- | 479.15552 | -1.28 | 179.06982,161.05917,133.06448 |
|  |  |  |  |  |  | [M-H]- | 195.06561 | -3.41 | 151.07568,137.06052,94.02876 |
| 133 | azelaic acid | 32.494 | C₉H₁₆O₄ | 123-99-9 | 188.10486 | [M+H]+ | 189.1113 | -4.45 | 145.10075,84.04473 |
|  |  |  |  |  |  | [M-H]- | 187.09676 | -4.39 | 169.08636,125.09616,97.06467 |
| 134 | tanshinol B | 32.495 | C₁₈H₁₆O₄ | 189290-30-0 | 296.10486 | [M+H]+ | 297.11188 | -0.87 | 279.06412,251.06937,166.06464 |
|  |  |  |  |  |  | [M-H]- | 295.09766 | 0.25 | 265.28713,220.06335,221.15407 |
| 135 | rosmarinic acid | 32.506 | C₁₈H₁₆O₈ | 20283-92-5 | 360.08452 | [M-H]- | 359.077 | -0.68 | 197.04498,179.03421,161.02357 |
| 136 | caffeic acid | 32.515 | C₉H₈O₄ | 331-39-5 | 180.04226 | [M+H]+ | 181.04874 | -4.44 | 165.03847,135.04375 |
| 137 | umbelliferone | 32.516 | C₉H₆O₃ | 93-35-6 | 162.03169 | [M+H]+ | 163.03816 | -4.99 | 145.02800,135.04373,117.03341 |
| 138 | vinyl amyl ketone | 32.592 | C₈H₁₄O | 4312-99-6 | 126.10447 | [M+H]+ | 127.11131 | -3.46 | 109.10122,85.07634,69.07042 |
| 139 | damulin A | 32.611 | C₄₂H₇₀O₁₃ | 1202868-74-3 | 782.48164 | [M+H]+ | 783.48505 | -4.95 | 441.37280,405.34912,85.02872 |
| 140 | psoromic acid | 32.689 | C₁₈H₁₄O₈ | 7299/11/8 | 358.06887 | [M+H]+ | 359.07443 | -4.78 | / |
|  |  |  |  |  |  | [M-H]- | 357.06177 | 0.49 | 225.05519,181.06519,121.02840 |
| 141 | salvianolic acid G | 32.703 | C₁₈H₁₂O₇ | 136112-79-3 | 340.0583 | [M+H]+ | 341.0639 | -4.93 | 295.05902,279.06396,249.05367 |
|  |  |  |  |  |  | [M-H]- | 339.05096 | -0.18 | 295.06073,280.03751 |
| 142 | isosafrole | 32.712 | C₁₀H₁₀O₂ | 120-58-1 | 162.06808 | [M+H]+ | 163.07465 | -4.38 | 145.02802,135.04370,117.03337 |
| 143 | salvianolic acid I | 32.714 | C₂₇H₂₂O₁₂ | 150072-80-3 | 538.11113 | [M-H]- | 537.10388 | 0.06 | 295.06116,185.02376,109.02837 |
| 144 | 4,5-dihydro-3-hydroxy-3-(1-oxobutyl)-1(3H)-isobenzofuranone | 32.742 | C₁₂H₁₄O₄ | 94530-82-2 | 222.08921 | [M+H]+ | 223.09558 | -4.07 | 177.09048,149.05934,99.04418 |
|  |  |  |  |  |  | [M-H]- | 221.0816 | -1.48 | 177.09143,149.02304 |
| 145 | secoisolariciresinol | 32.867 | C₂₀H₂₆O₆ | 29388-59-8 | 362.17294 | [M-H]- | 361.16574 | 0.34 | 197.04504,161.02364,72.99176 |
| 146 | lactiflorin | 33.003 | C₂₃H₂₆O₁₀ | 88623-95-4 | 462.1526 | [M+H]+ | 463.15784 | -4.7 | / |
|  |  |  |  |  |  | [M-H]- | 461.14517 | 0.17 | 339.10883,177.05498,121.02846 |
| 147 | majonoside R2 | 33.068 | C₄₁H₇₀O₁₄ | 81534-63-6 | 786.47656 | [M+H]+ | 787.48065 | -4.09 | / |
| 148 | dihydrokaempferol | 33.109 | C₁₅H₁₂O₆ | 480-20-6 | 288.06339 | [M+H]+ | 289.06924 | -4.93 | 24709315,169.01263,147.04355 |
|  |  |  |  |  |  | [M-H]- | 287.05585 | -0.92 | 181.01341,166.99789,153.01839 |
| 149 | safflomin C | 33.328 | C₃₀H₃₀O₁₄ | 126093-98-9 | 614.16356 | [M+H]+ | 615.16809 | -4.46 | 289.06949,169.01251,123.04388 |
|  |  |  |  |  |  | [M-H]- | 613.15582 | -0.74 | 359.41556,287.05685,119.04921 |
| 150 | roseoside | 33.338 | C₁₉H₃₀O₈ | 54835-70-0 | 386.19407 | [M+H]+ | 387.19989 | -3.76 | 295.05911,230.11687,139.03847 |
|  |  |  |  |  |  | [M-H]- | 385.18732 | 1.36 | 180.97250,112.98457 |
| 151 | 1,4-benzodioxane-6-carboxylic acid | 33.379 | C₉H₈O₄ | 4442-54-0 | 180.04226 | [M+H]+ | 181.04874 | -4.44 | 163.03844,139.03857,111.04409 |
| 152 | 6-hydroxykaempferol-3,6,7-triglucoside | 33.382 | C₃₃H₄₀O₂₂ | 145134-62-9 | 788.20112 | [M+H]+ | 789.20886 | 0.59 | 521.10559,323.05383,139.03857 |
|  |  |  |  |  |  | [M-H]- | 787.19421 | 0.47 | 625.14148,463.08762,301.03516 |
| 153 | 3-hydroxy-2,8-dimethyl-1,4-phenanthrenedione | 33.412 | C₁₆H₁₂O₃ | 65907-77-9 | 252.07864 | [M-H]- | 251.07097 | -1.58 | / |
| 154 | salvianolic acid Y | 33.417 | C₃₆H₃₀O₁₆ | 1638738-76-7 | 718.15338 | [M-H]- | 717.14575 | -0.5 | 519.09351,339.05124,321.04068 |
| 155 | baicalin | 33.685 | C₂₁H₁₈O₁₁ | 21967-41-9 | 446.08491 | [M+H]+ | 447.08997 | -4.98 | / |
|  |  |  |  |  |  | [M-H]- | 445.07712 | -1.16 | 269.04584,113.02315 |
| 156 | disporopsin | 33.723 | C₁₆H₁₄O₆ | 1430334-05-6 | 302.07904 | [M+H]+ | 303.08484 | -4.89 | 187.03839,159.04359 |
| 157 | galloylpaeoniflorin | 33.82 | C₃₀H₃₂O₁₅ | 122965-41-7 | 632.17412 | [M+H]+ | 633.17853 | -4.54 | / |
|  |  |  |  |  |  | [M-H]- | 631.16705 | 0.33 | 399.09274,313.05676,169.01346 |
| 158 | salvianolic acid A | 33.87 | C₂₆H₂₂O₁₀ | 96574-01-5 | 494.1213 | [M-H]- | 493.11389 | -0.26 | 295.06134,185.02379,109.02839 |
| 159 | senkyunolide R | 33.923 | C₁₂H₁₆O₅ | 172549-37-0 | 240.09977 | [M-H]- | 239.09196 | -2.22 | 195.10196,154.02643,94.02869 |
| 160 | salvianolic acid C | 33.947 | C₂₆H₂₀O₁₀ | 115841-09-3 | 492.10565 | [M-H]- | 491.09882 | 0.91 | 295.06122,185.02379,109.02838 |
| 161 | benzoyloxypaeoniflorin | 34.083 | C₃₀H₃₂O₁₃ | 72896-40-3 | 600.18429 | [M-H]- | 599.17765 | 1.1 | 342.14584,291.06430,137.02353 |
| 162 | neocurdione | 34.291 | C₁₅H₂₄O₂ | 108944-67-8 | 236.17763 | [M+H]+ | 237.18384 | -4.52 | 179.14235,161.13156,99.04427 |
| 163 | 9'''-methyllithosperMate B | 34.308 | C₃₇H₃₂O₁₆ | 1167424-31-8 | 732.16903 | [M-H]- | 731.16217 | 0.56 | 533.10889,353.06706,336.05619 |
| 164 | tanshindiol C | 34.318 | C₁₈H₁₆O₅ | 97465-71-9 | 312.09977 | [M-H]- | 311.09259 | 0.3 | 296.06927,252.07921,174.95508 |
| 165 | catechin gallate, (-)- | 34.338 | C₂₂H₁₈O₁₀ | 130405-40-2 | 442.09 | [M-H]- | 441.08078 | -4.39 | / |
| 166 | tanshinol A | 34.403 | C₁₈H₁₂O₄ | 189290-28-6 | 292.07356 | [M+H]+ | 293.08051 | -1.13 | 147.04362,130.04955,84.04465 |
|  |  |  |  |  |  | [M-H]- | 291.06625 | -0.1 | / |
| 167 | ginsenoside Ra1 | 34.52 | C₅₈H₉₈O₂₆ | 83459-41-0 | 1210.63463 | [M+H]+ | 1211.63708 | -4.86 | / |
| 168 | 3-tert-butyladipic acid | 34.582 | C₁₀H₁₈O₄ | 10347-88-3 | 202.12051 | [M+H]+ | 203.12694 | -4.19 | 187.03824,149.02290,116.05273 |
|  |  |  |  |  |  | [M-H]- | 201.11264 | -2.93 | 183.10188,139.11191 |
| 169 | N1,N5,N10-(E)-tri-p-coumaroylspermidine | 34.642 | C₃₄H₃₇N₃O₆ | 131086-78-7 | 583.26824 | [M-H]- | 582.26105 | 0.15 | 462.20374,342.14612,119.04916 |
| 170 | polygonatoside A | 34.659 | C₃₃H₅₂O₈ | 14144-06-0 | 576.36622 | [M+H]+ | 577.37103 | -4.27 | / |
| 171 | gypenoside XVII | 34.75 | C₄₈H₈₂O₁₈ | 80321-69-3 | 946.55012 | [M+H]+ | 947.55292 | -4.73 | 407.36581,145.04913,85.02874 |
| 172 | quercetin | 34.83 | C₁₅H₁₀O₇ | 117-39-5 | 302.04265 | [M+H]+ | 303.04843 | -4.96 | 256.09604,117.06986 |
|  |  |  |  |  |  | [M-H]- | 301.03525 | -0.42 | / |
| 173 | retinene | 34.873 | C₂₀H₂₈O | 116-31-4 | 284.21402 | [M+H]+ | 285.22028 | -3.58 | / |
| 174 | malonylginsenoside Rb1 | 34.944 | C₅₇H₉₄O₂₆ | 88140-34-5 | 1194.60333 | [M-H]- | 1193.5957 | -0.3 | 1149.60559,1107.59631,1150.60913 |
| 175 | benzyl cinnamate | 35.046 | C₁₆H₁₄O₂ | 103-41-3 | 238.09938 | [M-H]- | 237.09174 | -1.11 | 193.08646,131.04939 |
| 176 | salvigenin | 35.05 | C₁₈H₁₆O₆ | 19103-54-9 | 328.09469 | [M+H]+ | 329.10068 | -3.92 | 283.09503,269.07938,98.95426 |
|  |  |  |  |  |  | [M-H]- | 327.08774 | 1 | / |
| 177 | tanshinlactone | 35.056 | C₁₇H₁₂O₃ | 105351-70-0 | 264.07864 | [M+H]+ | 265.08481 | -4.21 | / |
| 178 | L(-)-verbenone | 35.059 | C₁₀H₁₄O | 1196-01-6 | 150.10447 | [M+H]+ | 151.11125 | -3.26 | 133.10075,108.51744,95.04928 |
| 179 | ginsenoside Rb3 | 35.121 | C₅₃H₉₀O₂₂ | 68406-26-8 | 1078.59237 | [M+H]+ | 1079.59534 | -4.01 | 407.36649,325.11166,127.03873 |
|  |  |  |  |  |  | [M-H]- | 1077.58057 | -0.24 | 783.49292,191.05542,89.02319 |
| 180 | ginsenoside Rg1 | 35.126 | C₄₂H₇₂O₁₄ | 22427-39-0 | 800.49221 | [M+H]+ | 801.49573 | -4.69 | 421.34406,143.10623 |
|  |  |  |  |  |  | [M-H]- | 799.48145 | -4.35 | / |
| 181 | 11-deoxyalisol B | 35.134 | C₃₀H₄₈O₃ | 155073-73-7 | 456.36035 | [M+H]+ | 457.36542 | -4.83 | / |
|  |  |  |  |  |  | [M-H]- | 455.3533 | 0.51 | / |
| 182 | acacetin-7-O-β-D-glucuronide | 35.147 | C₂₂H₂₀O₁₁ | 38226-83-4 | 460.10056 | [M+H]+ | 461.10562 | -4.82 | 285.07465,270.05148 |
|  |  |  |  |  |  | [M-H]- | 459.09366 | 0.81 | 283.06088,268.03790,113.02292 |
| 183 | queen bee acid | 35.231 | C₁₀H₁₈O₃ | 765-01-5 | 186.12559 | [M+H]+ | 187.13211 | -4.58 | 151.11148,109.06503,84.96012 |
| 184 | benzoylalbiflorin | 35.329 | C₃₀H₃₂O₁₂ | 184103-78-4 | 584.18938 | [M-H]- | 583.18188 | 0.17 | 335.05420,291.06375,121.02843 |
| 185 | malonylginsenoside Rb3 | 35.343 | C₅₆H₉₂O₂₅ | 1227255-74-4 | 1164.59277 | [M+H]+ | 1165.59558 | -3.83 | / |
|  |  |  |  |  |  | [M-H]- | 1163.58459 | -0.77 | 1077.58423,1059.57825,89.02316 |
| 186 | ginsenoside F5 | 35.543 | C₄₁H₇₀O₁₃ | 189513-26-6 | 770.48164 | [M-H]- | 769.47247 | 0.32 | 637.43268,475.37894,101.02318 |
| 187 | procyanidin B1 | 35.595 | C₃₀H₂₆O₁₂ | 20315-25-7 | 578.14243 | [M-H]- | 577.1355 | 0.6 | 193.05003,178.02647,163.03922 |
| 188 | mudanpioside J | 35.665 | C₃₁H₃₄O₁₄ | 262350-52-7 | 630.19486 | [M-H]- | 629.18768 | 0.17 | 557.88068,336.71585,121.02841 |
| 189 | cinnamic acid | 35.895 | C₉H₈O₂ | 621-82-9 | 148.05243 | [M+H]+ | 149.05908 | -4.21 | 131.04877,103.05428 |
| 190 | ferulaldehyde | 35.979 | C₁₀H₁₀O₃ | 20649-42-7 | 178.06299 | [M-H]- | 177.0549 | -4.58 | 162.03143,149.05997,118.96493 |
| 191 | przewaquinone B | 36.416 | C₁₈H₁₂O₄ | 76829-01-1 | 292.07356 | [M+H]+ | 293.07953 | -4.47 | 251.07010,223.07446,207.10077 |
| 192 | coniferyl ferulate | 36.432 | C₂₀H₂₀O₆ | 63644-62-2 | 356.12599 | [M-H]- | 355.11884 | 0.35 | 311.12909,296.10565,281.08228 |
| 193 | ginsenoside Rs1 | 36.472 | C₅₅H₉₂O₂₃ | 87733-67-3 | 1120.60294 | [M+H]+ | 1121.60607 | -4.16 | / |
| 194 | naringenin | 36.473 | C₁₅H₁₂O₅ | 480-41-1 | 272.06847 | [M+H]+ | 273.07455 | -4.39 | 153.01768,147.04355 |
|  |  |  |  |  |  | [M-H]- | 271.05887 | 0.53 | 151.00293,119.04916,107.01299 |
| 195 | apigenin | 36.473 | C₁₅H₁₀O₅ | 520-36-5 | 270.05282 | [M+H]+ | 271.06134 | -4.56 | 251.06923,223.07440,136.06129 |
|  |  |  |  |  |  | [M-H]- | 269.04568 | 0.51 | 225.05589,151.00298,119.04935 |
| 196 | benzyl glucopyranoside | 36.58 | C₁₃H₁₈O₆ | 4304/12/5 | 270.11034 | [M-H]- | 269.10318 | 0.44 | 151.00282,119.04920,107.01271 |
| 197 | sinapyl alcohol | 36.669 | C₁₁H₁₄O₄ | 537-33-7 | 210.08921 | [M+H]+ | 211.09544 | -4.96 | / |
|  |  |  |  |  |  | [M-H]- | 209.08131 | -2.94 | 191.07054,163.07513,121.02841 |
| 198 | o-octylphenol | 36.743 | C₁₄H₂₂O | 949-13-3 | 206.16707 | [M+H]+ | 207.1734 | -4.56 | 189.09042,135.11639,105.06989 |
| 199 | E-butylidenephthalide | 36.774 | C₁₂H₁₂O₂ | 76681-73-7 | 188.08373 | [M+H]+ | 189.09018 | -4.4 | 161.09538,143.0850891.05449 |
| 200 | kaempferol | 36.896 | C₁₅H₁₀O₆ | 520-18-3 | 286.04774 | [M+H]+ | 287.05368 | -4.66 | / |
|  |  |  |  |  |  | [M-H]- | 285.0405 | 0.12 | 168.23839,99.61977 |
| 201 | 9-oxo-10E,12Z-octadecadienoic acid | 37.083 | C₁₈H₃₀O₃ | 54232-59-6 | 294.21949 | [M+H]+ | 295.22546 | -4.44 | 279.06458,251.06932,223.07452 |
| 202 | stearidonic acid | 37.098 | C₁₈H₂₈O₂ | 20290-75-9 | 276.20893 | [M+H]+ | 277.21497 | -4.49 | 235.16721,149.02286,93.07015 |
| 203 | cnidilide | 37.103 | C₁₂H₁₈O₂ | 3674/3/1 | 194.13068 | [M+H]+ | 195.13719 | -3.94 | 163.03831,135.11644,81.07024 |
| 204 | undecylenic acid | 37.374 | C₁₁H₂₀O₂ | 112-38-9 | 184.14633 | [M-H]- | 183.13835 | -3.81 | / |
| 205 | trifolirhizin | 37.598 | C₂₂H₂₂O₁₀ | 6807-83-6 | 446.1213 | [M+H]+ | 447.12634 | -5 | 385.08684,237.07495,193.04880 |
|  |  |  |  |  |  | [M-H]- | 445.11389 | -0.29 | / |
| 206 | gypenoside Re | 37.66 | C₄₈H₈₂O₁₈ | 52286-59-6 | 946.55012 | [M+H]+ | 947.55328 | -4.39 | 407.36588,145.04910,85.02872 |
| 207 | γ-methoxyisoeugenol | 37.832 | C₁₁H₁₄O₃ | 63644-71-3 | 194.09429 | [M+H]+ | 195.10098 | -3.03 | 163.03850,131.97395,113.96362 |
|  |  |  |  |  |  | [M-H]- | 193.08633 | -3.52 | 149.05974,109.02836 |
| 208 | 1,4,5(6H)-phenanthrenetrione,7,8-dihydro-3-hydroxy-8,8-dimethyl-2-(1-methylethyl)- | 38.013 | C₁₉H₂₀O₄ | 125675-06-1 | 312.13616 | [M+H]+ | 313.14197 | -4.7 | 269.15250,199.07465,171.07994 |
| 209 | 3-phenylpropyl benzenepropanoate | 38.035 | C₁₈H₂₀O₂ | 60045-27-4 | 268.14633 | [M+H]+ | 269.15234 | -4.71 | 251.06842,223.07260,136.06128 |
| 210 | salvinolone | 38.37 | C₂₀H₂₆O₃ | 120278-22-0 | 314.18819 | [M+H]+ | 315.19394 | -4.88 | 269.19000,161.05939,133.06435 |
|  |  |  |  |  |  | [M-H]- | 313.18076 | -0.52 | 193.05006,161.02415,134.03638 |
| 211 | methyleugenol | 38.631 | C₁₁H₁₄O₂ | 93-15-2 | 178.09938 | [M+H]+ | 179.10608 | -3.24 | 123.04382,88.02117,56.96544 |
|  |  |  |  |  |  | [M-H]- | 177.09142 | -3.86 | 149.09616,118.96542 |
| 212 | paeonol | 39.562 | C₉H₁₀O₃ | 552-41-0 | 166.06299 | [M+H]+ | 167.06973 | -3.24 | 142.96643,102.97030,84.95998 |
| 213 | ramalic acid | 39.655 | C₁₈H₁₈O₇ | 500-37-8 | 346.10525 | [M+H]+ | 347.11096 | -4.53 | 305.07712,284.29364,137.05919 |
|  |  |  |  |  |  | [M-H]- | 345.09802 | 0.13 | 194.02229,155.03391,140.01062 |
| 214 | N-butylidenephthalide | 39.655 | C₁₂H₁₂O₂ | 551-08-6 | 188.08373 | [M+H]+ | 189.09018 | -4.4 | 171.07985,133.02802,117.06976 |
| 215 | nortanshinone | 39.861 | C₁₇H₁₂O₄ | 97399-70-7 | 280.07356 | [M+H]+ | 281.07962 | -4.34 | 149.02290,57.07057 |
|  |  |  |  |  |  | [M-H]- | 279.06622 | -0.21 | 251.07118,235.07687,117.03349 |
| 216 | 3-N-butylphthalide | 39.878 | C₁₂H₁₄O₂ | 6066-49-5 | 190.09938 | [M+H]+ | 191.10583 | -4.32 | 149.05920,135.04367,91.05447 |
|  |  |  |  |  |  | [M-H]- | 189.09148 | -3.3 | / |
| 217 | 1(3H)-isobenzofuranone,3-butyl-4,5-dihydro-4-hydroxy-, (3S,4R)- | 39.884 | C₁₂H₁₆O₃ | 114569-33-4 | 208.10994 | [M+H]+ | 209.11624 | -4.71 | 191.10594,149.05914,135.05914 |
|  |  |  |  |  |  | [M-H]- | 207.1021 | -2.74 | 163.11203,121.02876 |
| 218 | 8-methoxypsoralen | 40.038 | C₁₂H₈O₄ | 298-81-7 | 216.04226 | [M+H]+ | 217.04865 | -4.12 | / |
| 219 | palbinone | 40.046 | C₂₂H₃₀O₄ | 139954-00-0 | 358.21441 | [M-H]- | 357.20703 | -0.28 | 197.04478,161.02359,135.04407 |
| 220 | (7aS)-4,4,7a-trimethyl-6,7-dihydro-5H-benzofuran-2-one | 40.283 | C₁₁H₁₆O₂ | 81800-41-1 | 180.11503 | [M+H]+ | 181.12161 | -3.85 | 163.11127,135.11647,116.97182 |
|  |  |  |  |  |  | [M-H]- | 179.10701 | -4.13 | 168.96689,135.04443,112.98457 |
| 221 | 4-N-heptylphenol | 40.433 | C₁₃H₂₀O | 1987-50-4 | 192.15142 | [M+H]+ | 193.15805 | -3.34 | 177.16327,160.59045,95.08569 |
| 222 | δ-decalactone | 40.513 | C₁₀H₁₈O₂ | 705-86-2 | 170.13068 | [M+H]+ | 171.1373 | -3.87 | 146.96085,128.95062,89.07124 |
|  |  |  |  |  |  | [M-H]- | 169.12265 | -4.43 | / |
| 223 | senkyunolide F | 40.595 | C₁₂H₁₄O₃ | 94530-84-4 | 206.09429 | [M-H]- | 205.08641 | -2.95 | 161.09633,132.05693,106.04124 |
| 224 | (+)-ledol | 40.786 | C₁₅H₂₆O | 577-27-5 | 222.19837 | [M+H]+ | 223.20461 | -4.67 | 194.11684,138.05452,110.06001 |
| 225 | 1,2,15,16-tetrahydrotanshiquinone | 40.872 | C₁₈H₁₆O₃ | 126979-84-8 | 280.10994 | [M+H]+ | 281.11627 | -3.39 | 149.02289,57.07053 |
| 226 | 6-(1-oxopentyl)-1-cyclohexene-1-carboxylic acid | 41.301 | C₁₂H₁₈O₃ | 6697-07-0 | 210.12559 | [M-H]- | 209.11771 | -2.91 | 165.12785,121.02834 |
| 227 | ethyl 3-phenylpropanoate | 41.385 | C₁₁H₁₄O₂ | 2021-28-5 | 178.09938 | [M+H]+ | 179.10608 | -3.24 | 137.05939,116.00182,88.02180 |
| 228 | dehydroandrographolide | 41.405 | C₂₀H₂₈O₄ | 134418-28-3 | 332.19876 | [M-H]- | 331.19165 | 0.51 | 287.16428,283.17041,112.98427 |
| 229 | vomifoliol | 41.784 | C₁₃H₂₀O₃ | 23526-45-6 | 224.14124 | [M+H]+ | 225.14761 | -4.05 | / |
|  |  |  |  |  |  | [M-H]- | 223.13345 | -2.29 | / |
| 230 | benzyl benzoate | 41.898 | C₁₄H₁₂O₂ | 120-51-4 | 212.08373 | [M+H]+ | 213.09039 | -2.89 | 128.02686,140.99998,95.04935 |
| 231 | (Z)-3-(2-hydroxybutylidene)isobenzofuran-1(3H)-one | 42.126 | C₁₂H₁₂O₃ | 94530-83-3 | 204.07864 | [M+H]+ | 205.08519 | -3.58 | 187.07480,159.07982,149.02272 |
|  |  |  |  |  |  | [M-H]- | 203.07063 | -3.6 | 174.03183,160.01584 |
| 232 | octadecanedioic acid | 42.155 | C₁₈H₃₄O₄ | 871-70-5 | 314.24571 | [M+H]+ | 315.25156 | -4.55 | 287.04279,279.03177,81.07022 |
|  |  |  |  |  |  | [M-H]- | 313.23865 | 0.68 | 183.13853,129.09158 |
| 233 | alpha-Linolenic acid | 42.163 | C₁₈H₃₀O₂ | 68424-45-3 | 278.22458 | [M+H]+ | 279.23059 | -4.55 | 149.02296,98.08578,81.07025 |
| 234 | inermin | 42.24 | C₁₆H₁₂O₅ | 19908-48-6 | 284.06847 | [M+H]+ | 285.07449 | -4.42 | 270.05115,229.08508,187.03841 |
|  |  |  |  |  |  | [M-H]- | 283.061 | -0.68 | 268.03787,163.00320,95.38589 |
| 235 | phenanthro[3,2-b]furan-7,11-dione, 8,9-dihydro-2-hydroxy-4,8-dimethyl- | 42.248 | C₁₈H₁₄O₄ | 128397-97-7 | 294.08921 | [M+H]+ | 295.09528 | -4.61 | 277.08987,249.05987,185.05879 |
|  |  |  |  |  |  | [M-H]- | 293.08188 | -0.18 | 265.08704,250.06354,89.02297 |
| 236 | methyl rosmarinate | 42.446 | C₁₉H₁₈O₈ | 99353-00-1 | 374.10017 | [M+H]+ | 375.10571 | -4.63 | / |
|  |  |  |  |  |  | [M-H]- | 373.09299 | 0.26 | 179.03426,135.04425 |
| 237 | neocryptotanshinone | 42.596 | C₁₉H₂₂O₄ | 109664-02-0 | 314.15181 | [M+H]+ | 315.15756 | -4.85 | 251.10536,213.09007 |
| 238 | matairesinol | 42.944 | C₂₀H₂₂O₆ | 580-72-3 | 358.14164 | [M-H]- | 357.13467 | 0.87 | 253.12460,197.04524,161.02361 |
| 239 | isocryptotanshinone | 42.985 | C₁₉H₂₀O₃ | 22550-15-8 | 296.14124 | [M+H]+ | 297.14713 | -4.71 | 279.06403,251.06949,223.07465 |
| 240 | isobornyl acetate | 43.284 | C₁₂H₂₀O₂ | 125-12-2 | 196.14633 | [M+H]+ | 197.15283 | -3.95 | 181.12181,153.12689,74.09691 |
|  |  |  |  |  |  | [M-H]- | 195.13844 | -3.11 | 176.89453,136.23328 |
| 241 | ginsenoyne C | 43.354 | C₁₇H₂₄O₃ | 138828-84-9 | 276.17254 | [M+H]+ | 277.17856 | -4.57 | 235.16830,179.10599 |
| 242 | 3-(2,4-dihydroxyphenyl)propionice acid | 43.359 | C₉H₁₀O₄ | 5631-68-5 | 182.05791 | [M+H]+ | 183.06444 | -4.11 | 131.97389,116.97176,56.96544 |
|  |  |  |  |  |  | [M-H]- | 181.04974 | -4.88 | 149.02359,135.04431,112.98455 |
| 243 | methylcinnamate | 43.464 | C₁₀H₁₀O₂ | 103-26-4 | 162.06808 | [M+H]+ | 163.07465 | -4.38 | 131.04883,103.05432 |
| 244 | dillapiole | 43.534 | C₁₂H₁₄O₄ | 484-31-1 | 222.08921 | [M+H]+ | 223.09549 | -4.77 | 177.05402,149.02286 |
|  |  |  |  |  |  | [M-H]- | 221.0816 | -1.48 | 177.09140,149.09628,87.04391 |
| 245 | 20-Deoxocarnosol | 43.558 | C₂₀H₂₆O₅ | 80225-53-2 | 346.17802 | [M-H]- | 345.17029 | -1.33 | 209.04642,106.76741 |
| 246 | epi-spiroketallactone | 43.826 | C₁₇H₁₆O₃ | 113472-19-8 | 268.10994 | [M+H]+ | 269.11612 | -4.11 | 251.10553,223.07503,213.05377 |
|  |  |  |  |  |  | [M-H]- | 267.10269 | 0.09 | / |
| 247 | procyanidin A2 | 43.932 | C₃₀H₂₄O₁₂ | 41743-41-3 | 576.12678 | [M+H]+ | 577.13123 | -4.91 | / |
|  |  |  |  |  |  | [M-H]- | 575.11755 | -3.38 | 553.29480,221.06647,101.02319 |
| 248 | 20(R)-ginsenoside Rg2 | 44.42 | C₄₂H₇₂O₁₃ | 80952-72-3 | 784.49729 | [M+H]+ | 785.50159 | -4.39 | 680.51190,145.04919,85.02874 |
|  |  |  |  |  |  | [M-H]- | 783.48688 | 0.43 | 763.40179,475.37869,101.02334 |
| 249 | ccinnamyl cinnamate | 44.543 | C₁₈H₁₆O₂ | 122-69-0 | 264.11503 | [M+H]+ | 265.12119 | -4.24 | 209.11699,57.07056 |
| 250 | deoxyneocryptotanshinone | 44.549 | C₁₉H₂₂O₃ | 27468-20-8 | 298.15689 | [M+H]+ | 299.16284 | -4.46 | 198.06303,121.06465 |
| 251 | ailanthoidol | 44.557 | C₁₉H₁₈O₅ | 156398-61-7 | 326.11542 | [M-H]- | 325.10831 | 0.49 | 281.11847,265.08722,253.12334 |
| 252 | estrone | 44.655 | C₁₈H₂₂O₂ | 53-16-7 | 270.16198 | [M+H]+ | 271.16824 | -3.75 | / |
| 253 | senkyunolide A | 44.779 | C₁₂H₁₆O₂ | 63038-10-8 | 192.11503 | [M+H]+ | 193.12149 | -4.25 | 175.11101,147.11638,137.05928 |
| 254 | benzyl alcohol | 44.816 | C₇H₈O | 100-51-6 | 108.05751 | [M+H]+ | 109.0647 | -0.87 | 87.00432,71.06087,54.03452 |
| 255 | 2'-hydroxyacetophenone | 44.817 | C₈H₈O₂ | 118-93-4 | 136.05243 | [M+H]+ | 137.0592 | -3.69 | 95.08570,81.07025 |
| 256 | pyrethrin II | 44.858 | C₂₂H₂₈O₅ | 121-29-9 | 372.19367 | [M-H]- | 371.18646 | 0.18 | / |

**Note.** / : not detected.

**Supplementary Table S3.** The compound-Targets gene network constructed using core genes and corresponding compounds

| **NO.** | **Compounds** | **Targets** |
| --- | --- | --- |
| 1 | (+)-ledol | ESR1 |
| 2 | (7aS)-4,4,7a-trimethyl-6,7-dihydro-5H-benzofuran-2-one | PPARG |
| 3 | (Z)-3-(2-hydroxybutylidene)isobenzofuran-1(3H)-one | PTGS2, MMP9 |
| 4 | 1(3H)-isobenzofuranone,3-butyl-4,5-dihydro-4-hydroxy-, (3S,4R)- | PTGS2 |
| 5 | 1,2,15,16-tetrahydrotanshiquinone | STAT3, EGFR, CASP3 |
| 6 | 11-deoxyalisol B | PTGS2, MTOR, EGFR, CCND1 |
| 7 | 1-galloyl-glucose | HSP90AA1 |
| 8 | 1-hydroxypinoresinol 1-O-glucoside | TNF, HIF1A |
| 9 | 20(R)-ginsenoside Rg2 | STAT3 |
| 10 | 20-Deoxocarnosol | SRC, MTOR, EGFR, CCND1, CASP3 |
| 11 | 2-coumarate | TLR4, MMP9 |
| 12 | 2-hydroxy-3’,4’- dihydroxyacetophenone | ALB |
| 13 | 2'-hydroxyacetophenone | ALB |
| 14 | 3-(2,4-dihydroxyphenyl)propionice acid | EGFR |
| 15 | 3,5-dimethyl-p-anisic acid | PTGS2 |
| 16 | 3-hydroxy-2,8-dimethyl-1,4-phenanthrenedione | PTGS2, PPARG |
| 17 | 3-hydroxysenkyunolide A | JUN |
| 18 | 3-phenylpropyl benzenepropanoate | AKT1 |
| 19 | 4-hydroxy-3,5-dimethoxycinnamic acid | TLR4, STAT3, PTGS2, MMP9, ESR1, EGFR, CTNNB1 |
| 20 | 4-hydroxycinnamic acid | TLR4, MMP9, ESR1 |
| 21 | 4-N-heptylphenol | SRC, PTGS2, ESR1 |
| 22 | 4-O-β-D-glucopyranosyloxy-benzoic acid | HSP90AA1 |
| 23 | 6-(1-oxopentyl)-1-cyclohexene-1-carboxylic acid | TNF, PTGS2, PPARG, MAPK3, HSP90AA1, ESR1, CXCL8 |
| 24 | 6-hydroxykaempferol 3,6-diglucoside | TNF, PTGS2 |
| 25 | 6-hydroxykaempferol-3,6,7-triglucoside | TNF, PTGS2 |
| 26 | 8-debenzoylpaeoniflorin | STAT3, HSP90AA1 |
| 27 | 8-methoxypsoralen | NFKB1, EGFR |
| 28 | 9'''-methyllithosperMate B | MMP9, CASP3 |
| 29 | 9-oxo-10E,12Z-octadecadienoic acid | PTGS2, PPARG, HIF1A, ESR1 |
| 30 | acacetin-7-O-β-D-glucuronide | TNF, PTGS2, EGFR |
| 31 | albiflorin | SRC, MMP9, HSP90AA1, EGFR |
| 32 | alpha-Linolenic acid | PTGS2, PPARG, MAPK3, IL6, ESR1 |
| 33 | apigenin | SRC, PTGS2, MMP9, ESR1, EGFR, AKT1 |
| 34 | baicalin | TNF, EGFR |
| 35 | benzoylalbiflorin | MMP9, HSP90AA1, GAPDH, CASP3 |
| 36 | benzoyloxypaeoniflorin | HSP90AA1 |
| 37 | benzyl cinnamate | STAT3 |
| 38 | caffeic acid | TLR4, STAT3, MMP9, ESR1, EGFR |
| 39 | catechin gallate, (-)- | STAT1, MMP9, BCL2 |
| 40 | ccinnamyl cinnamate | MMP9 |
| 41 | cianidanol | STAT1, SRC, MMP9, HIF1A, ESR1, EGFR, BCL2 |
| 42 | cinnamaldehyde | TLR4 |
| 43 | cinnamic acid | TLR4, MMP9, EGFR |
| 44 | cinnamyl alcohol | TLR4 |
| 45 | damulin A | STAT3 |
| 46 | dehydroandrographolide | PTGS2, EGFR, CCND1 |
| 47 | dihydrocaffeic acid | EGFR |
| 48 | dihydrokaempferol | STAT1, SRC, PPARG, HIF1A, ESR1, BCL2 |
| 49 | dillapiole | HSP90AA1, EGFR, AKT1 |
| 50 | dimethyl lithospermate B | MMP9, EGFR |
| 51 | dimethyl succinate | TLR4 |
| 52 | disporopsin | SRC, PTGS2, PPARG, JUN, FOS, ESR1 |
| 53 | epi-spiroketallactone | SRC, HIF1A, EGFR, CCND1 |
| 54 | estrone | SRC, PTGS2, MMP9, ESR1 |
| 55 | ethyl gallate | SRC |
| 56 | ferulic Acid | TLR4, STAT3, PTGS2, MMP9, EGFR, CCND1 |
| 57 | galloylpaeoniflorin | HSP90AA1 |
| 58 | ginsenoside F1 | STAT3, HSP90AA1 |
| 59 | ginsenoside F5 | STAT3 |
| 60 | ginsenoside Rg1 | STAT3, HSP90AA1 |
| 61 | ginsenoside Rs1 | GAPDH, CASP3 |
| 62 | hexenal | MMP9, AKT1 |
| 63 | hyperin | TNF, PTGS2 |
| 64 | inermin | SRC |
| 65 | isocryptotanshinone | PTGS2 |
| 66 | isomaltopaeoniflorin | HSP90AA1 |
| 67 | isoquercitrin | TNF, PTGS2 |
| 68 | isorhamnetin | SRC, MMP9, EGFR, AKT1 |
| 69 | kaempferol | SRC, PTGS2, MMP9, ESR1, EGFR, AKT1 |
| 70 | L(-)-verbenone | MAPK3, ESR1 |
| 71 | lactiflorin | HSP90AA1, EGFR |
| 72 | leucoside | TNF, PTGS2 |
| 73 | majonoside R2 | STAT3, HSP90AA1 |
| 74 | methyl 2-aminobenzoate | STAT3, HSP90AA1 |
| 75 | methyl gallate | SRC |
| 76 | methyl rosmarinate | STAT3, MTOR, MMP9, ESR1, EGFR, BCL2 |
| 77 | methylcinnamate | TLR4 |
| 78 | mudanpioside E | HSP90AA1 |
| 79 | mudanpioside J | HSP90AA1 |
| 80 | multiflorin B | TNF, PTGS2 |
| 81 | myristicin | SRC, MMP9 |
| 82 | N1,N5,N10-(E)-tri-p-coumaroylspermidine | MMP9, EGFR |
| 83 | N-acetyl-2-oxopropanamide | CASP3 |
| 84 | naringenin | SRC, PPARG, MMP9, ESR1, BCL2 |
| 85 | N-butylidenephthalide | PTGS2 |
| 86 | neocurdione | PPARG |
| 87 | nortanshinone | ESR1 |
| 88 | o-octylphenol | PTGS2, ESR1, CCND1 |
| 89 | oxypaeoniflora | HSP90AA1 |
| 90 | oxypeucedanin | HSP90AA1, CASP3 |
| 91 | paeoniflorin | HSP90AA1 |
| 92 | paeonilactone A | PTGS2, NFKBIA, EGFR |
| 93 | paeonilactone B | PTGS2, EGFR, CASP3 |
| 94 | paeonoside | MMP9, GAPDH, ESR1, EGFR, CCND1 |
| 95 | palbinone | ESR1 |
| 96 | phenanthro[3,2-b]furan-7,11-dione, 8,9-dihydro-2-hydroxy-4,8-dimethyl- | TNF, SRC, PTGS2, MTOR, MMP9, ESR1, EGFR, CCND1, CCND1, CCND1, CASP3 |
| 97 | phenol，4-ethyl- | ESR1 |
| 98 | polygonatoside A | STAT3 |
| 99 | procyanidin A2 | MMP9 |
| 100 | procyanidin B1 | SRC, MMP9, HIF1A, EGFR, BCL2 |
| 101 | propyl gallate | SRC, MMP9 |
| 102 | protocatechuic acid | ALB |
| 103 | przewaquinone B | MTOR, IL6, HIF1A, EGFR, CASP3 |
| 104 | pyrethrin II | PPARG |
| 105 | pyrogallol | EGFR |
| 106 | quercetin | SRC, MMP9, EGFR, AKT1 |
| 107 | ramalic acid | MMP9, CTNNB1 |
| 108 | rhodionin | TNF |
| 109 | riboflavin | MMP9, GAPDH |
| 110 | rosmarinic acid | MMP9, ESR1, EGFR |
| 111 | rutin | TNF, PTGS2 |
| 112 | salicylic acid | ALB |
| 113 | salvianic acid A | ESR1, EGFR |
| 114 | salvianolic acid A | MMP9, ALB |
| 115 | salvianolic acid C | JUN, CASP3 |
| 116 | salvianolic acid D | MMP9, CASP3 |
| 117 | salvianolic acid F | PTGS2, MMP9 |
| 118 | salvianolic acid G | MMP9, EGFR, CASP3 |
| 119 | salvianolic acid I | MMP9 |
| 120 | salvigenin | SRC, PTGS2, MMP9, ESR1, EGFR |
| 121 | salvinolone | TGFB1, MTOR, BCL2 |
| 122 | scutellarein | SRC, PTGS2, MMP9, ESR1, EGFR, AKT1 |
| 123 | senkyunolide A | IL1B |
| 124 | senkyunolide F | PTGS2, EGFR |
| 125 | senkyunolide I | STAT3, MMP9, JUN |
| 126 | senkyunolide R | STAT3, CASP3 |
| 127 | shanzhiside methyl ester | HSP90AA1 |
| 128 | sinapyl alcohol | PTGS2, MMP9, MAPK3, IL6, ESR1, EGFR |
| 129 | stearidonic acid | TP53, PPARG, MMP9, BCL2 |
| 130 | sweroside | GAPDH, EGFR |
| 131 | tanshindiol C | EGFR, CASP3 |
| 132 | tanshinol A | STAT3, MTOR, MMP9, JUN, HSP90AA1, EGFR, CASP3 |
| 133 | tanshinol B | EGFR, CASP3 |
| 134 | tanshinone ⅡB | SRC, EGFR |
| 135 | tinctormine | CASP3 |
| 136 | trifolirhizin | PTGS2 |
| 137 | trijuganone C | TLR4, STAT3, SRC, EGFR |
| 138 | undecylenic acid | PTGS2, PPARG, CXCL8 |
| 139 | vanillic acid | MMP9 |
| 140 | vomifoliol | TNF |
| 141 | vulgarin | PTGS2, PPARG, AKT1 |
| 142 | δ-decalactone | EGFR |

**Supplementary Table S4.** Predicted binding energies (kcal/mol) of the top 10 GXJC compounds with TNF, eNOS, and ET-1 targets.

| **NO.** | **Compounds** | TNF | eNOS | ET-1 |
| --- | --- | --- | --- | --- |
| 128 | sinapyl alcohol | -4.6 | -6.6 | -4.4 |
| 122 | scutellarein | -9.0 | -9.1 | -5.4 |
| 76 | methyl rosmarinate | -6.6 | -8.8 | -4.7 |
| 69 | kaempferol | -8.8 | -9.3 | -4.5 |
| 132 | tanshinol A | -6.3 | -8.3 | -5.0 |
| 52 | disporopsin | -8.7 | -8.3 | -4.9 |
| 41 | Cianidanol | -9.1 | -8.6 | -5.3 |
| 23 | 6-(1-oxopentyl)-1-cyclohexene-1-carboxylic acid | -4.8 | -7.2 | -3.5 |
| 19 | 4-hydroxy-3,5-dimethoxycinnamic acid | -4.8 | -7.0 | -4.2 |
| 96 | phenanthro[3,2-b]furan-7,11-dione, 8,9-dihydro-2-hydroxy-4,8-dimethyl-  (Dihydroisotanshinone I) | -11.4 | -15.0 | -9.2 |
